# Properties, Preparation and Applications of Low Dimensional Transition Metal Dichalcogenides

**DOI:** 10.3390/nano8070463

**Published:** 2018-06-26

**Authors:** Lei Yang, Chenggen Xie, Juncheng Jin, Rai Nauman Ali, Chao Feng, Ping Liu, Bin Xiang

**Affiliations:** 1 Key Laboratory of Biomimetic Sensor and Detecting Technology of Anhui Province, School of Materials and Chemical Engineering, West Anhui University, Lu’an 237012, China; cgxie@wxc.edu.cn (C.X.); jjc@wxc.edu.cn (J.J.); 2 Department of Materials Science & Engineering, CAS key Lab of Materials for Energy Conversion, Synergetic Innovation Center of Quantum Information & Quantum Physics, University of Science and Technology of China, Hefei 230026, China; nauman@mail.ustc.edu.cn (R.N.A.); fengchao901008@163.com (C.F.); 15209835824@163.com (P.L.)

**Keywords:** low dimensional transition metal dichalcogenides, crystal structure, electronic structure, preparation methods, chemical vapor deposition

## Abstract

Low-dimensional layered transition metal dichalcogenides (TMDs) have recently emerged as an important fundamental research material because of their unique structural, physical and chemical properties. These novel properties make these TMDs a suitable candidate in numerous potential applications. In this review, we briefly summarize the properties of low-dimensional TMDs, and then focus on the various methods used in their preparation. The use of TMDs in electronic devices, optoelectronic devices, electrocatalysts, biosystems, and hydrogen storage is also explored. The cutting-edge future development probabilities of these materials and numerous research challenges are also outlined in this review.

## 1. Introduction

Since graphene prepared by the mechanical exfoliation method was first discovered in 2004 [1], it sparked a research boom of other kinds of analogous layered structured materials [2,3,4,5,6,7,8,9,10,11]. The layered transition metal dichalcogenides (TMDs) is an important kind of layered research material [12,13,14,15,16,17]. The generalized formula of layered TMDs is MX_2_, where X represents a chalcogen e.g., S, Se, Te, and M represents the transition metal atoms in Group 4–7 and in part of Group 8–10 [18], such as Mo, W, Ti, Hf, Re, etc. In each layer of TMDs, the metal atoms are sandwiched by chalcogen atoms with strong chalcogen-metal covalent bond interactions [19]. However, the interlayers stacking is coupled by weak van der Waals forces, which enables the easy exfoliation of bulk crystals into the two-dimensional morphology [18,19]. Similar to graphene, TMDs also display interesting layer-dependent properties, when their thickness decreases from three dimensions to two. For example, bulk MoS_2_ is an indirect band gap material with a band gap of 1.3 eV [20]. However, monolayer MoS_2_, noticeably different from the bulk, is a direct band gap material with a band gap of 1.9 eV [21]. This indirect-to-direct band gap transition results in the dramatic enhancement of photoluminescence (PL), which can be attributed to quantum confinement and surface effects [19,21,22]. In monolayer TMDs, the lack of inversion symmetry combined with spin-orbit coupling leads to coupled spin and valley physics, which makes it possible to control the spin and valley in these monolayered materials; this doesn’t exist in multilayer ones [23,24]. Meanwhile, the experiment has realized the optical pumping of a single valley (and spin) with circular polarized light [25,26]. As the dimensions of two-dimensional (2D) TMDs are reduced to one-dimensional (1D) and zero-dimensional (0D), the edge effects become much more prominent [27,28,29]. As predicated by theoretical studies, the electronic structures and properties, together with the quantum confinement effects of these 1D TMDs nanoribbons/nanobelts and 0D TMDs quantum dots (QDs), dramatically changed [27,28,29]. 

These novel properties promote the exploration of new methods to prepare the low-dimensional TMDs. Numerous methods are available for their synthesis—mechanical exfoliation [30,31], solution-based exfoliation [29,32,33,34], Li-intercalation exfoliation [35,36], laser and plasma-induced thinning [37,38], hydrothermal method [39,40] chemical vapor deposition (CVD) [41,42,43,44,45,46,47,48,49,50], annealing of the (NH_4_)_2_MoS_4_ precursor [51,52], chemical vapor transport [53,54], and so on. Driven by advanced preparation methods and increased research interests, applications based on low-dimensional TMDs have enabled scientists to conquer the appealing properties of these materials. Take MoS_2_ as an example—the room temperature on/off ratio of monolayer MoS_2_ exceeds 10^8^, and in-plane carrier mobility can reach 200–500 cm^2^/V·s [31]. Furthermore, low-dimensional TMDs have shown excellent electrocatalytic performance in water splitting [55,56,57,58].

Several review papers have summarized the structures and properties of two-dimensional TMDs as well as their applications, such as energy storage and conversion, optoelectronic, electrocatalysis, electrochemical biosensors, and so on [59,60,61,62,63,64,65,66,67,68,69]. In this paper, the focus is mainly on the preparation of low-dimensional TMDs, including 2D thin layers, 1D nanoribbons/nanobelts/nanotubes, and 0D QDs. We initially induce the properties of low-dimensional TMDs. The recent progress of various methods to prepare such materials is then explored. Sequentially, their achieved progress for applications in electronic devices, optoelectronic devices, electrocatalysts, hydrogen storage and biosystems is also discussed. In the end, we summarize possible future research directions and potential challenges.

## 2. Properties and Characterization

### 2.1. Crystal Structure

**Figure 1:**
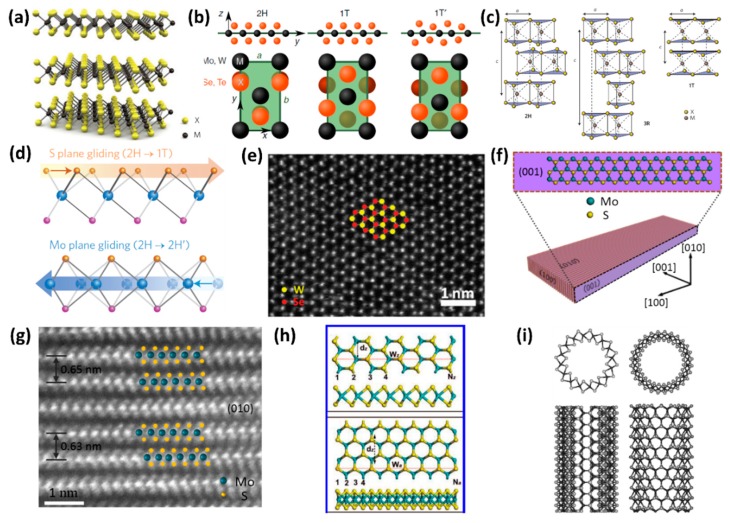
Structure of low-dimensional TMDs. (**a**) Three-dimensional schematic representation of a typical MX_2_ structure. The yellow balls and grey balls refer to chalcogen atoms and transition metal atoms, respectively [70]. (**b**) The layer structure (**top**) and rectangular unit cell (**down**) of 2H, 1T and 1T’ phases. The 2H phase refer to trigonal prismatic structure, 1T and 1T’are called octahedral and distorted octahedral, respectively [71]. (**c**) Schematic structure of 2H, 3R and 1T phase of MX_2_. The interlayer spacing is ~6.5 Å and the stacking index c indicates the number of layers in each stacking order [70]. (**d**) Schematic illustration of the gliding of S plane (**top**) and Mo plane (**bottom**), which result in phase transition. The S plane glide results in a 2H → 1T phase transition, and Mo plane glide results in a 2H → 2H’ transition, where the 2H’ phase is a 60° rotational phase of 2H [73]. (**e**) HRSTEM image of monolayer WSe_2_ with defect-free atomic lattices. The W and Se atoms form the hexagonal ring with different brightness as denoted by the color cartoon spheres [43]. (**f**,**g**) Schematic illustration and HRSTEM image of MoS_2_ nanobelts. The vertical atomic layers form the nanobelt structure, and these layer edges form the top surface of the nanobelt [80]. (**h**) Side and top views of the structures of 8-zigzag MoS_2_ nanoribbon (**top**) and 15-armchair MoS_2_ nanoribbon (**down**). The W_z_ (W_a_) and d_z_ (d_a_) correspond to the ribbon width and 1-D unit cell distance, respectively [27]. (**i**) Armchair (8, 8) MoS_2_ nanotube (**left**) and zigzag (14, 0) MoS_2_ nanotube (**right**). The dark and light atoms are Mo and S, respectively [81]. Reproduced with permission from [27]. Copyright American Chemical Society, 2008. Reproduced with permission from [43]. Copyright The Royal Society of Chemistry, 2015. Reproduced with permission from [70]. Copyright Macmillan Publishers Limited, 2012. Reproduced with permission from [71,73]. Copyright Macmillan Publishers Limited, 2014. Reproduced with permission from [80]. Copyright American Chemical Society, 2015. Reproduced with permission from [81]. Copyright The American Physical Society, 2000.

Bulk-layered TMDs are composed of X-M-X sandwich layers with interlayer spacing of ~6.5 Å [31,70], as shown in Figure 1a. With different coordination spheres of the transition metal atoms, each individual sandwich layer exhibits several structural phases in which the trigonal prismatic (2H, the prefix ‘2’ is irrelevant in monolayers) or octahedral (1T) is commonly observed, as shown in Figure 1b [59,71]. The Mo atoms in 2H phase are prismatically coordinated to six surrounding S atoms. However, in the 1T phase, six S atoms form a distorted octahedron around one transition metal atom [19,70]. From a side view, the 2H phase is characterized by an AbA stacking order and the 1T phase corresponds to an AbC stacking order, where the capital and lower letters correspond to metal and chalcogen atomic planes, respectively. With different polymorphs and stacking sequence, the bulk TMDs present three polytypes: 1T, 2H and 3R, where the digits correspond to the number of layers in the stacking sequence, and the letters stand for trigonal, hexagonal and rhombohedral, respectively [18]. The schematic structures are shown in Figure 1c [70]. In bulk TMDs formed by group VI transition metals (metal = Mo or W; chalcogen = S, Se or Te, except WTe_2_), 2H is a thermodynamically stable phase and 1T is a metastable phase [59]. For example, in MoS_2_, the molybdenite is commonly found in 2H phase [18]. The 1T-MoS_2_ is a metastable metallic phase, which will undergo a first order phase transition and transform into the thermodynamically stable form of 2H-MoS_2_ when the temperature is higher than 100 °C [72]. Suenaga’s group systematically studied the structural transformation between the semiconducting (2H) and metallic (1T) phases in atomic resolution by using in situ scanning transmission electron microscopy (TEM) technique [73]. Their results indicated that the 2H/1T phase transition involved intra layer sulphur and/or molybdenum atomic plane gliding and required an intermediate phase (α-phase) as a precursor (Figure 1d) [73]. Moreover, the 1T-MoS_2_ is often prepared by the Li intercalation method [36,72,74]. Recently, Zhang’s group also synthesized 1T’-MoX_2_ (X = S, Se) crystals with lateral size up to hundreds of micrometers. The 1T’ is a lower-symmetry phase, which is a distorted version of the 1T structure as shown in Figure 1b [71,75]. The 1T’-MoX_2_ (X = S, Se) is a metastable phase, which is convertible to the 2H-MoS_2_ phase after thermal annealing or laser irradiation. Similar to the 2H phase, the 3R phase shows semiconducting behavior and often exists in the synthetic MoS_2_ [18,76]. In contrast to traditional 2H-Mo(W)S_2_ and Mo(W)Se_2_, the group IV and V TMDs compounds such as TiS_2_ and TaSe_2_ are observed in 1T phase under ambient conditions [71,77]. However, for the WTe_2,_ the 1T’ phase is more stable than its hexagonal-phase structure, according to theoretical calculations and experimental results [78,79].

For the edge structure of group VI TMDs compounds, such as MoS_2_, it is better to expose two types of low index edge terminations: the (10$\bar{1}$0) Mo edge and the ($\bar{1}$010) S edge [82,83,84]. In the internal of MoS_2_ “sandwich” layers, the atoms are all saturated [82,85]. Theoretical calculations indicate that the Mo edges became unstable when the Mo bonds on the edge are unsaturated [82,85]. The edge can be the structure with one S (50% coverage) or two S atoms (100% coverage) per Mo edge atom (called S dimers) due to the fact that the edges have no perfect trigonal prismatic coordination [82,85]. In this condition, the Mo atoms bonding with six S atoms are saturated.

High-resolution scanning TEM (HRSTEM) is an efficient characterization approach to investigate the crystal structure of low-dimensional layered TMDs at the atomic level. Especially in single layer TMDs, it is easy to distinguish M sites and X-X sites [46,72,86,87]. In HRSTEM imaging, the brightness of the atom is proportional to the mean square of the atom’s number along the direction of the electron beam [88]. For example, in monolayer WSe_2,_ the W and Se atoms form a hexagonal ring with different brightness as denoted by the color cartoon spheres in Figure 1e [43]. The brighter dots correspond to the W sites, while the darker dots refer to the Se-Se sites (the atomic number of W and Se atom is 74 and 34, respectively). The atomic number of S is 16, which is smaller than that of Se. If one or two S atoms replace Se-Se sites, the brightness will become darker than that of original Se-Se sites. Therefore, HRSTEM can also be employed to portray the distribution of the chalcogen atoms in the monolayer MX_2_ alloy system.

When the dimensions of 2D TMDs materials are reduced, one-dimensional TMDs, such as nanoribbons, nanobelts or nanotubes, are obtained. Recently, our group synthesized single-crystal atomic-layered MoS_2_ nanobelts for the first time [80]. HRSTEM characterization indicated that the (001) basal planes of the MoS_2_ nanobelts were vertical on the substrate (Figure 1f,g) and the edges of the base planes formed the top surfaces of the nanobelts. Xu et al. also achieved a similar structure, but the MoS_2_ nanobelts showed a polycrystalline structure [49]. For TMDs nanoribbons, they usually present “belt” morphologies with the c-axis of base planes vertically aligned to the substrate [27,89,90,91,92]. The edges of TMDs nanoribbons were predominant due to its large surface-to-volume ratio. In general, the TMDs nanoribbons usually show zigzag- or armchair-terminated edges, which make the TMDs show different structure-related properties. Figure 1h shows the top and side views of the structure of 8-zigzag-MoS_2_ nanoribbon (top) and (b) 15-armchair-MoS_2_ nanoribbon (bottom) [27]. The W_z_ (W_a_) and d_z_ (d_a_) correspond to the ribbon width and 1-D unit cell distance, respectively.

Similar to carbon nanotubes, tubular structures can be analogously constructed by twining a 2D triple layer of TMDs around the surface of a cylinder, thus rolling up the sheets along specific directions in the 2D lattice [81,93]. By describing the nanotubes in terms of the primitive 2D lattice vectors and two integer indices $\vec{B}=n\vec{a}+m\vec{b}$, the nanotube could be divided into three classes: *n* = *m* “armchair” nanotubes, *n* ≠ 0, *m* = 0 “zigzag” nanotubes, and *n* ≠ *m* “chiral nanotubes” [81]. Figure 1i schematically illustrates the armchair (8, 8) MoS_2_ nanotube and zigzag (14, 0) MoS_2_ nanotube; a triple layer of atoms created a wall that exhibits certain “roughness” on the outer shell [81].

Further limiting the dimension of the 1D materials, TMDs with small planar dimensions, namely QDs, are obtained. The size of TMDs QDs could be as small as ~2 nm, which contain fewer than 50 molecules, and the thickness could be as small as a triple layer of atoms [94]. Compared to nanosheets, QDs have higher special surface area. Unlike other QDs with dangling bonds on the surface, TMDs QDs have no dangling bonds on their basal planes, except at possible defect sites and edges [29].

### 2.2. Band Structure and Optical Properties

**Figure 2:**
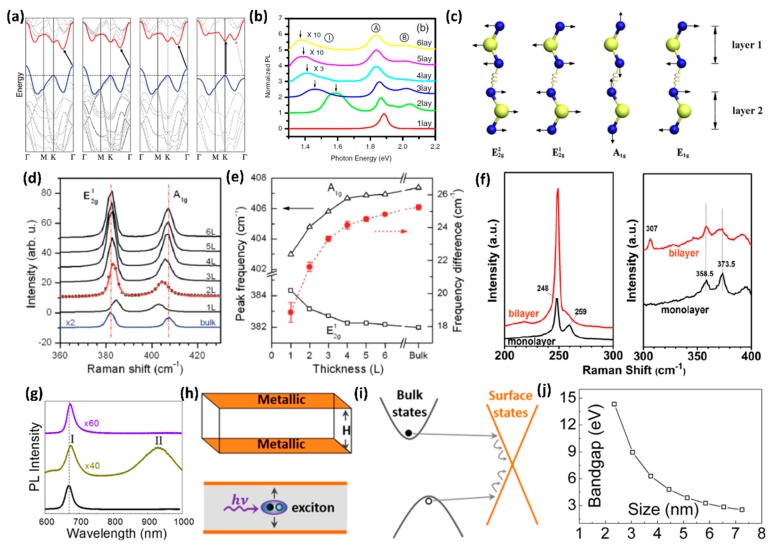
Band structure and optical properties of low-dimensional TMDs. (**a**) Calculated band structures of bulk MoS_2_, quadrilayer MoS_2_, bilayer MoS_2_ and monolayer MoS_2_. Bulk MoS_2_, quadrilayer MoS_2_, bilayer MoS_2_ are all indirect band gap materials. When the thickness is reduced, the indirect band gap becomes larger. It becomes direct band gap when the thickness reaches the 2D limit [95]. (**b**) PL spectra of MoS_2_ with different thickness (1–6 layers). Peak I corresponds to the indirect band gap transition, while peaks A and B refer to the direct band gap transition [21]. (**c**) Schematic view of the atomic displacements of the four Raman-active modes [39]. (**d**) Raman spectra of MoS_2_ with different thickness. With increasing the layer from 1 to 6, the $E_{2g}^{1}$ shows red shifts, and *A*_1*g*_ shows blue shifts [96]. (**e**) The layer dependent of the $E_{2g}^{1}$ and *A*_1*g*_ Raman modes (**left vertical axis**) and the difference of $E_{2g}^{1}$ and *A*_1*g*_ (**right vertical axis**) as a function of layer thickness [96]. (**f**) Raman spectra of monolayer and bilayer WSe_2_. For bilayer WSe_2_, there was an obvious peak at 307 cm^−1^ which was absent in monolayer one [97]. (**g**) PL spectra of the nanobelt, an exfoliated multilayer, and a CVD-grown monolayer. Compared with exfoliated multilayer, the indirect band gap transition peak nearly disappeared [80]. (**h**,**i**) Scheme of the structure and electronic band picture for the nanobelt. The induced excitons first diffuse to the metallic surface and then nonradiatively decay [80]. (**j**) The relationship between the MoS_2_ QDs size and bandgap. With reducing the QDs size, reduced band gap was obtained [29]. Reproduced with permission from [21]. Copyright The American Physical Society, 2010. Reproduced with permission from [29]. Copyright AIP Publishing LLC, 2015. Reproduced with permission from [39]. Copyright Macmillan Publishers Limited, 2014. Reproduced with permission from [80]. Copyright American Chemical Society, 2015. Reproduced with permission from [95,96]. Copyright American Chemical Society, 2010. Reproduced with permission from [97]. Copyright American Chemical Society, 2013.

The various chemical compositions and structural phases make the TMDs a promising candidate for a broad range of electronic properties. Here, we discuss the semi-conductive TMDs formed by group VI transition metals Mo and W, combined with S and Se. In the bulk form of layered TMDs, the conduction band minimum is located at halfway between Γ and K points in Brillouin zone. However, the valence band maximum is located at Γ point, and the valence band sub-maximum and the conduction band sub-minimum coincide at K point [12,21]. By decreasing thickness, the direct excitonic transition energy at K point does not change, while the indirect band gap increases monotonically [95]. The direct excitonic transition energy becomes larger than the indirect one, when the thickness of TMDs is decreased to the 2D limit, which makes the monolayer TMDs transform into the direct band gap semiconductor (Figure 2a) [95]. It has been established that the energy states of the conduction band at K point mainly originated from the strongly localized d orbitals of Mo atoms, which had minimal interlayer coupling [95]. The states near the Γ point and the point of indirect band gap, however, came from the combination of d orbitals on Mo atoms and antibonding p_z_ orbitals on S atoms. This is the reason why the band gap energies are sensitive to layer thickness.

PL measurement is an effective characterization method that reflects the evolution of electronic structure of TMDs with various thicknesses. For instance, the PL spectra intensity of bulk MoS_2_ can be neglected. When the thickness is decreased to a few layers, like 2–6 layers, the MoS_2_ samples display multiple emission peaks, as shown in Figure 2b [21]. Peaks labeled A and B correspond to the direct excitonic transitions at the Brillouin zone K point, while the Peak I corresponds to the indirect band gap transition [21]. It is obvious that the PL intensity becomes much stronger when the thickness is reduced to the monolayer; this is mainly due to the fact that the band gap is transformed from indirect one to the direct one [19,21,22]. Surprisingly, when the lateral dimensions of metallic 2D TMDs are reduced, similar PL responses are observed [98,99]. This might be due to the spatial quantum confinement effect of the electron clouds, similar to observations in other metallic nanoparticles [98]. Besides the PL spectra, the Raman spectra are also relevant to the thickness of layered TMDs and can be used to characterize their electronic properties [96,97,100]. Figure 2c shows the atomic displacements of the four Raman-active modes of MoS_2_ or WSe_2_ [39]. In MoS_2_, the *E*_1*g*_ and *E*^2^_2*g*_ are hardly observed due to selection rules and limited rejection of the Rayleigh scattering [22,26,96]. For the out of-plane *A*_1*g*_ mode, the restoring force is mainly from the interlayer van der Waals interaction [101]. With the increase of the number of layers, the restoring forces are enhanced, so that the *A*_1*g*_ mode frequency is increased. The in-plane $E_{2g}^{1}$ mode is a symmetric mode. When the number of layers are increased, the frequency shifts to the lower region. The decrease in frequency is possibly due to the long range Columbic interactions from the coupled dipoles induced from Mo-S bonds [101,102]. Figure 2d shows the representative Raman spectra for monolayer, few-layer (2–6 layers) and bulk MoS_2_ samples. With increasing sample thickness, the blue shifts of *A*_1*g*_ and red shifts of $E_{2g}^{1}$ are observed. When the thickness is increased to six layers, the frequencies of $E_{2g}^{1}$ and *A*_1*g*_ modes converge to bulk values [96]. By measuring the frequency difference of $E_{2g}^{1}$ and *A*_1*g*_ active modes, we can verify the thickness of few layers MoS_2_ sample. Figure 2e shows the frequencies of $E_{2g}^{1}\;$and *A*_1*g*_ Raman modes (left vertical axis) and their difference (right vertical axis) with layer thickness [96]. The frequencies and their difference are also summarized in Table 1. Compared with MoS_2_, some second order and combinational modes, like the peak around 307 cm^−1^, are also observed in the WSe_2_ bulk crystal, as shown in Figure 2f [97]. Instead of the $E_{2g}^{1}$ and *A*_1*g*_ active modes, this second order mode peak can be used to confirm its monolayer configuration, because it is absent in monolayer configuration [97,103,104].

For 1D TMDs nanoribbons, the edges are predominant. First-principles computations indicate that the zigzag MoS_2_ nanoribbons show metallic behavior, irrespective of ribbon width and thickness [27]. Whereas, the armchair MoS_2_ nanoribbons are semiconducting and the band gap is converted to a constant value of 0.56 eV with increasing ribbon width [27]. Furthermore, the zigzag MoS_2_ nanoribbons are more stable than the armchair one [27]. This stable metallic edges were verified in the MoS_2_ nanobelts experimentally, as mentioned in Section 2.1 [80]. In MoS_2_ nanobelts, the PL intensity of direct band gap transition peak was as weak as that in the exfoliated multilayer, which indicated that the nanobelt structure was still an indirect band gap material. However, the indirect band gap transition peak disappeared, as shown in Figure 2g [80]. The disappearing of the indirect band gap transition peak was caused by full metallic edge states, which provided an effective channel for exciton decay. The top surface was fully composed of metallic edge states, as shown in Figure 2h [80]. Therefore, the excitons (electron-hole pairs) could decay non-radiatively from the edge states (Figure 2i), and the indirect band gap PL peak nearly disappeared. As for nanotubes such as MoS_2_, the armchair (*n*, *n*) nanotubes exhibit a nonzero moderate direct gap and the zigzag (*n*, 0) nanotubes possess a narrow direct band gap [81]. Furthermore, the (*n*, *n*) tubes show a small indirect gap similar to the direct gap of (*n*, 0) nanotubes [81].

Different from the 2D and 1D TMDs, the band gap of TMDs QDs have shown size-dependent properties due to quantum confinement. For MoS_2_ QDs, The PL peak position indicated a blue-shift as compared to big MoS_2_ sheets [29,105]. The smaller the size is, the larger the energy gap will be [29,105]. The band gap of MoS_2_ QDs versus particle size is shown in Figure 2j [29]. According to the effective-mass approximation, the correlation between the band gap and size can be derived by the following equation:

$$\;E=E_{g}+\frac{h^{2}}{8\mu r^{2}}-\frac{1.8e^{2}}{4\varepsilon_{0}\varepsilon r}$$

where μ is the reduced mass of exciton with value of 0.16 $m_{0}$ ($m_{0}$ is the free-electron mass), h is the Plank’s constant, *E_g_* is the indirect band gap value of 1.29 eV, and the dielectric constant is about 6.8.

### 2.3. Band Gap Engineering

Engineering the electronic structure of layered TMDs is an efficient way to tailor their physical and chemical properties, and is also of great importance to broaden their applications. In the beginning, alloying was an effective approach to tune electronic structures. Xie’s group initially realized the tunable band gap in Mo_1−*x*_W*_x_*S_2_ monolayers [106]. By varying the W content in the alloys, the band gap was continuously tuned from 1.82 eV to 1.99 eV, confirmed by the PL spectra [106]. Their results indicated that the band gap energy of monolayer Mo_1−*x*_W*_x_*S_2_ was smaller than the linear combination of that of MoS_2_ and WS_2_. That is to say, it agreed with the bowing effect as observed in many bulk semiconductor alloys: E(PL,Mo_1−*x*_W*_x_*S_2_) < (1 − *x*)E(PL,MoS_2_) + *x*E(PL,WS_2_) [106]. Our group successfully synthesized the monolayer MoS_2(1−*x*)_Se_2*x*_ and WS_2(1−*x*)_Se_2*x*_ alloys by using the CVD method, and realized the 0.31 eV and 0.4 eV band gap tuning, respectively [107,108,109]. 

**Figure 3:**
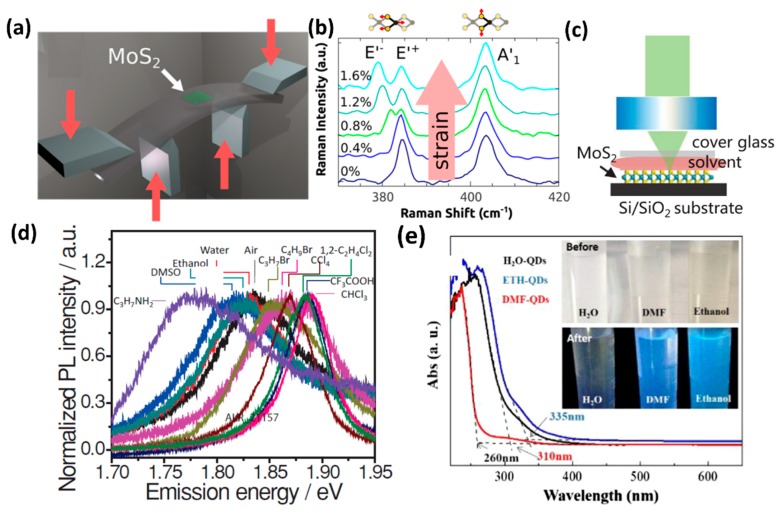
Band gap engineering of low-dimensional TMDs. (**a**) Schematic illustration of the bending apparatus used to exert strain on MoS_2_. By controllably bending the polycarbonate beam of the four-point bending apparatus, a uniaxial strain was exerted on monolayer and bilayer MoS_2_ [110]. (**b**) Evolution of the Raman spectra with strain ranged from 0 to 1.6%. With increased strain, the symmetry of the crystal broke, which made the degenerate E’ peak split into two sub-peaks [110]. (**c**) Schematic depiction of the micro-Raman-PL experimental configuration. The surface of the monolayer MoS_2_ is covered by different solvents during experiments [113]. (**d**) PL spectra of monolayer MoS_2_ with different solvent surroundings. The emission peaks were tuned in the range of 1.78–1.90 eV. Red shifts of the monolayer MoS_2_ PL peaks were observed when the surroundings changed from air to non-halogenated solvents (water, ethanol, dimethyl sulfoxide, propylamine). Blue shifts were observed when the surroundings changed from air to halogenated solvents (trifluoroacetic acid, methylene chloride, chloroform, carbon tetrachloride, butyl bromide, propyl bromide) [113]. (**e**) UV-visible absorption of functionalized MoS_2_ QDs. The absorption edges of MoS_2_ QDs in DI water (H_2_O-QDs), in DMF and in ethanol were located at ~310 nm, ~260 nm, and ~335 nm, respectively [40]. Reproduced with permission from [40]. Copyright Elsevier Inc., 2017. Reproduced with permission from [110]. Copyright American Chemical Society, 2013. Reproduced with permission from [113]. Copyright Wiley-VCH Verlag GmbH & Co. KGaA, 2013.

Exerting strain is another useful way to engineer electronic structures [84,85,86]. As TMDs can resist large strains before they break, lattice strain engineering is an important strategy to tune their band gap [110,111,112]. Conley et al. applied uniaxial tensile mechanical strain on monolayer and bilayer MoS_2_ to investigate the evolution of their band structure and phonon spectra by using a four-point bending apparatus [112]. The schematic diagram is shown in Figure 3a [111]. By controlling the bend of the substrate, different uniaxial strains were applied to MoS_2_ [111]. Measured by PL spectroscopy, they found that the decrease in optical band gap was approximately linear with the strain [111]. The band gap tuning was ∼45 meV per 1% strain for monolayer MoS_2_ and ∼120 meV per 1% strain for bilayer MoS_2_. At the same time, they also found that the strain caused the phonon vibration modes to shift to lower frequencies and the degenerate E’ peak to split into two sub-peaks, as shown in Figure 3b. This was mainly due to the fact that the strain broke the symmetry of the crystal [111]. 

Recently, experimental results have also demonstrated that the controlled strain of 2D semiconductors is achieved during CVD growth by utilizing the thermal coefficient of expansion (TCE) mismatch between the substrate and the semiconductor [114]. For example, the TCE of WSe_2_ was 9.5 ± 3.2 ppm, which was much larger than that of aluminum nitride and fused silica with the value of 0.55 and 5.5 ppm, respectively. Therefore, aluminum nitride and fused silica could induce tensile strain on the as-grown WSe_2_. The strontium titanate had a TCE of 12 ppm, which yielded compressively strained samples and sapphire had a TCE value closely matched to WSe_2_, which could produce relaxed samples. After the growth at high temperature, substrates with stable built-in strains ranging from 1% tensile to 0.2% compressive were achieved. PL characterizations indicated that there was strain-induced indirect-to-direct optical transition in the directly-grown WSe_2_ bilayer and brightening of the dark exciton in monolayer WSe_2_ [114]. 

The strain effect could also influence the band gap of TMDs nanotubes and nanoribbons. For example, when the axial tensile strain was exerted on MoS_2_ nanotubes, the band gap would decrease for both armchair and zigzag nanotubes [93]. On increasing the axial tensile strain, progressive decrease in band gap was observed. For the armchair nanotubes, however, a semiconductor-to-metal transition happened when the axial tensile strain reached about 8% [93]. Meanwhile, an applied strain could also reduce the band gap of MoS_2_ nanoribbons [115].

Besides the strain and alloying, surrounding solvents can also influence the PL spectra of layered TMDs. Xie’s group investigated the effect of surrounding solvents on the PL of monolayer MoS_2_ [113]. Figure 3c shows a schematic experimental configuration in which the top surface of the MoS_2_ monolayer is covered by different solvents during experiments [113]. With different solvent surroundings, the PL spectra of MoS_2_ monolayers showed emission peak in the range of 1.78 to 1.90 eV, as shown in Figure 3d [113]. Their results suggested that the PL spectra of monolayer MoS_2_ showed blue shifts (up to 60 meV) with halogenated solvent surroundings (trifluoroacetic acid, methylene chloride, chloroform, carbon tetrachloride, butyl bromide, propyl bromide) and red shifts (up to −60 meV) with non-halogenated solvent surroundings (water, ethanol, dimethyl sulfoxide, propylamine), comparing to the PL spectra in air (Figure 3d) [113]. For the red shifts with non-halogenated solvents, it meant more stabilization for the excited state in polar solvents. However, for the halogenated solvents, the special bond between the halogen atoms and the MoS_2_ dissociated the negative trions to neutral excitons, which caused an abnormal blue shift of PL spectra [113].

For the MoS_2_ nanoribbons, edge passivation was also an effective way to engineer its band structure, because it could fluctuate the edge charge distribution. For example, in armchair MoS_2_ nanoribbons, the indirect band gap would be transferred to direct band gap when the edges were passivated by full hydrogen, amidogen or partial hydroxyl [90]. Compared with the bare armchair MoS_2_ nanoribbons or the hydrogen passivated MoS_2_ nanoribbons, the hydrogen and oxygen hybrid edge-terminated structure was more stable [89]. Different from the band gap of bare armchair MoS_2_ nanoribbons (~0.61 eV), the band gap of nanoribbons with hydrogen passivation and with hydrogen and oxygen hybrid passivation were about 0.60 eV and 1.43 eV, respectively [88]. When the armchair MoS_2_ nanoribbons were passivated by hydrogen and fluorine (nitrogen, phosphorus) hybrid, the nanoribbons showed metallic behavior [89]. 

Furthermore, surface passivation was also valid for band gap tuning of TMDs QDs. Our group realized a band gap tunability in MoS_2_ QDs passivated by different functional groups [40]. As shown in Figure 3e, the UV-visible absorption spectra characterization indicated that the absorption edge of MoS_2_ QDs in DI water was located at ~310 nm, while the absorption edge of MoS_2_ QDs dispersed in DMF and ethanol were located at ~260 nm and ~335 nm, respectively. Compared with MoS_2_ QDs in ethanol, there was a blue shift of 75 nm (1 eV) observed in the band gap of QDs in DMF. In general, electron-donating and electron-accepting behaviors occurred between the host material and functional groups. When dispersed in ethanol, MoS_2_ QDs were mainly functionalized by the hydroxyl groups, which could increase the capacity of the conjugate system by giving π electrons to the host material. The acceptance of the electrons raised the energy level of the highest-occupied molecular orbital of the host material MoS_2_ QDs, leading to a decreased band gap. When dispersed in DMF, MoS_2_ QDs were mainly functionalized by the aldehyde groups, which could reduce the energy level location of the highest occupied molecular orbital of the host material by withdrawing the electrons from the host material. As a result, an increased band gap was achieved. On varying the concentration of ethanol in distilled water, the band gap of MoS_2_ QDs showed a concentration-dependent phenomena: a red shift of ~100 meV on increasing the concentration of ethanol from 10% to 100%. This was caused by the fact that the number of hydroxyl groups in the functionalized ethanol-MoS_2_ QDs was different, as varied from the concentration.

An electric field could also be used to modulate the band gap of armchair MoS_2_ nanoribbons [116]. Theoretical calculations indicated that the band gap of monolayer armchair MoS_2_ nanoribbons could be significantly reduced and closed by a transverse field [116]. However, when the perpendicular field was applied, the band gap modulation was absent. On increasing the ribbon width, the critical strength of the transverse field for gap closure decreased. In contrast, in multilayer armchair MoS_2_ nanoribbons, the band gap can be effectively reduced by both transverse and perpendicular fields [116].

### 2.4. Mechanical Properties

**Figure 4:**
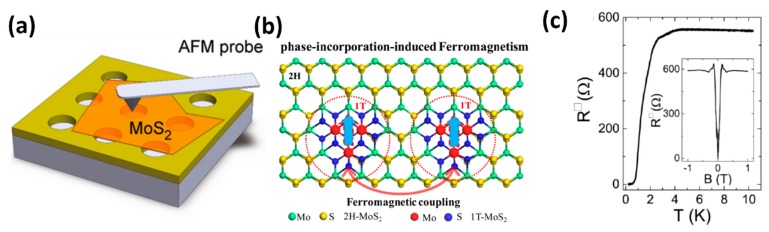
Mechanical, ferromagnetic and superconductivity properties of 2D TMDs. (**a**) Schematic illustration for the stiffness measurements of monolayer MoS_2_. During measurements, the AFM tip was placed above the center of the suspend area and slowly lowered while monitoring the deflection [117]. (**b**) Diagrammatic representation of the phase incorporation strategy to achieve ferromagnetism of 2H-MoS_2_ nanosheets. By incorporating the 1T-MoS_2_ phase into the 2H-MoS_2_ matrix, ferromagnetism was induced [118]. (**c**) Temperature dependence of the square resistance of ionic-gated WS_2_ FET at V_G_ = 3.7 V. The resistance decrease was observed with an onset at ~4 K, and the resistance reached zero at Tc ~ 0.5 K. The inset shows the magnetic field dependence of the square resistance at T = 0.25 K, which indicated that the WS_2_ reached the normal state value with magnetic field B ~ 0.14 T [119]. Reproduced with permission from [117]. Copyright American Chemical Society, 2011. Reproduced with permission from [118,119]. Copyright American Chemical Society, 2015.

For the application of bendable electronics in the future, the mechanical properties of layered TMDs should also be characterized. Kis’s group investigated the stretching and breaking of monolayer and bilayer MoS_2_ by using the nanoindentation in an atomic force microscope (AFM) [117]. During the measurement, the AFM tip was placed above the suspended MoS_2_ sample, which was transferred to the pre-patterned SiO_2_ substrate containing an array of 550 nm circular holes. The schematic illustration is shown in Figure 4a [117]. They found that the in-plane stiffness of monolayer MoS_2_ was 180 ± 60 N/m, corresponding to an effective Young’s modulus of 270 ± 100 GPa. This value of Young’s modulus was comparable to that of steel. They also observed that the break of MoS_2_ appeared at the average strength of 23 GPa, about 11% of its Young’s modulus, which meant that the sample was highly crystalline, almost defect-free, and could be used in many high-demand mechanical-related applications [117].

Similarly, Kaplan-Ashiri et al. also investigated the mechanical behavior of individual WS_2_ nanotubes by attaching the WS_2_ nanotube to a commercial silicon AFM cantilever [120]. The cantilever was placed in an AFM and the WS_2_ nanotube tip was pushed against the sputtered titanium surface, thus applying a controlled and measurable force. By using the Euler buckling formula, the average Young’s modulus of an individual WS_2_ nanotube was found to be 171 GPa, which was comparable to that of the bulk material (150 GPa). They also performed First-principle calculations to study the Young’s modulus of MoS_2_ nanotubes. The calculated results indicated that the armchair (*n*, *n*) nanotubes had a considerably smaller Young’s modulus than the zigzag (*n*, 0) nanotubes with a similar radius. By increasing the radius, the Young’s modulus of armchair nanotubes increased; however, the Young’s modulus of zigzag nanotubes remained essentially invariant with the tube’s radius and was very close to that of bulk MoS_2_ (238 GPa). Ataca et al. also calculated the mechanical properties of MoS_2_ nanoribbons [121]. For armchair MoS_2_ nanoribbons (*n* = 12) and zigzag MoS_2_ nanoribbons (*n* = 6), the calculated in-plane stiffness was 108.47 and 103.71 N/m, respectively. The difference between the stiffness values of armchair and zigzag nanoribbon was due to the different bonds and edge directions.

### 2.5. Ferromagnetic Properties

The spin-splitting property make the layered TMDs suitable for spintronics applications [70,122]. However, their applications in spintronics were hampered by their nonmagnetic property. If the room temperature ferromagnetism was endowed to nonmagnetic TMDs materials, the TMDs could be expected to act as an ideal spintronics channel material for nanodevices. Therefore, developing approaches to effectively induce magnetism in TMDs is highly desired. It is remarkable to achieve magnetism in nonmagnetic MoS_2_ without magnetic atom doping. In 2007, Zhang et al. synthesized edge-oriented MoS_2_ nanosheet-like films by thermal evaporation of the single-source precursor tetrakis (diethylaminodithiocarbomate) molybdate(IV) [123]. Magnetic characterization indicated that MoS_2_ nanofilms exhibited weak magnetism (~1–2 emu/g) with a Curie temperature of 685 K. Similar phenomena was also observed in MoS_2_ nanobelts [49]. Density functional theory (DFT) calculations demonstrated that magnetism mainly arose from unsaturated atoms at the edge sites. To get a better understanding of magnetism at the edge sites, Li et al. carried out first-principles computations to predict the magnetic properties of MoS_2_ nanoribbons with either zigzag- or armchair-terminated edges [27]. For zigzag nanoribbons, they showed that the ferromagnetic and magnetic moment mainly resulted from unsaturated edge atoms. In contrast, armchair nanoribbons were nonmagnetic. The ratio of edge atoms vs. total atoms was very important for magnetic behavior in MoS_2_ nanoribbons with zigzag- terminated edges. The magnetism of MoS_2_ zigzag nanoribbons became weaker as the ribbon width increased and disappeared in the infinitely single-layered MoS_2_ and bulk. This is the reason why nonmagnetic property is observed in common MoS_2_.

Besides the zigzag-terminated edges, doping defects on the base plane was also an effective way to induce and manipulate the magnetism of MoS_2_ nanosheets [124]. Recently, Wei’s Group realized ferromagnetism in MoS_2_ at room temperature by using a phase incorporation strategy [118]. During the synthesis, they intentionally introduced sulfur vacancies in 2H-MoS_2_ nanosheet by a two-step hydrothermal method. Figure 4b shows the schematic representation of the phase incorporation strategy to achieve ferromagnetism of 2H-MoS_2_ nanosheets. The sulfur vacancies could facilitate the transformation of the surrounding 2H-MoS_2_ into a 1T-MoS_2_ which greatly enhanced the saturation magnetization of 2H-MoS_2_ nanosheets at 300 K from 0.02 to 0.25 μB/Mo. Experimental characterizations and calculation results indicated that the ferromagnetism of this incorporated structure originated from exchange interactions between sulfur vacancies and Mo^4+^ ions [118].

### 2.6. Superconductivity

Superconductivity is another interesting property that exists in TMDs. Group V TMDs compounds like NbSe_2_ and TaS_2_ exhibit intrinsic superconductivity, which has been well elucidated in Ref. [59]. For semiconducting layered TMDs represented by 2H-MoS_2_, WS_2_ or MoSe_2_, the realization of low-resistance metallic behavior was the prerequisite to achieving superconductivity [59]. Doped by intercalations was an efficient way to increase electron concentration [125,126,127]. In bulk MoS_2_, when the metals were inserted into the adjacent MoS_2_ layers, the intercalation would donate the electrons to MoS_2_ and make the system metallic [128]. When MoS_2_ was intercalated with calcium and strontium, the Ca*_x_*MoS_2_ and Sr*_x_*MoS_2_ compounds started to superconduct at ~4 K and 5.6 K [125]. In the intercalated and metallic Rb_0.3_MoS_2_, superconductivity was discovered with a maximum T_c_ ~ 6.9 K [127]. However, doped by intercalation would cause structural modifications in the material and therefore might affect its electronic band structure.

Recently, TMDs based field effect transistors (FET) with a gate dielectric made of ionic liquid, LaF_3_ or KClO_4_/polyethylene glycol became a very promising technique, because this gate dielectric could be regarded as the nanometer-scale capacitor and potentially give rise to a giant capacitance [129,130,131]. When a voltage was applied to the electrolyte, the electron would accumulate at the surface and the density would become very large. For the MoS_2_, MoSe_2_, WS_2_ and MoTe_2_ the densities could be closed to or larger than 10^14^ cm^−1^ [119,128,129]. Upon cooling, electrical transportation indicated that the samples exhibited metallic behavior with the surface resistivity decreasing dramatically. For example, in WS_2,_ the decrease in resistance was observed with an onset at ~4 K and the resistance reached zero at T_c_ ~ 0.5 K (critical temperature), as shown in Figure 4c [119]. As we know, the application of a perpendicular magnetic field B would destruct the zero-resistance state. The inset of Figure 4c shows the magnetic field dependence of the square resistance at T = 0.25 K, which indicated that the WS_2_ reached the normal state value with magnetic field B ~ 0.14 T (critical magnetic field B_c_) [119]. As for MoS_2_, the electric-field-induced superconductivity was observed at T_c_ ~ 9.4 K, and the magnetic field of 3T broke the superconducting transition [128]. Shi et al. also studied the superconductivity of MoSe_2_, MoTe_2_ and WS_2_; their results indicated that the T_c_ was ~ 7.1 K, 2.8 K and 8.6 K, respectively [129]. To investigate the dependence of superconductivity on thickness, Costanzo et al. studied the evolution of gate-induced superconductivity in exfoliated MoS_2_ multilayers ranging from bulk-like to individual monolayers [132]. When the thickness was reduced from six to two layer, T_c_ and B_c_ decreased slowly. On reducing the thickness to monolayer, T_c_ and B_c_ were unambiguously suppressed as compared to all thicker multilayers. T_c_ jumped from approximately 7 K to 2 K and B_c_ became more than one order of magnitude smaller when passing from bilayer to monolayer MoS_2_. The suppression of superconductivity in thinner sample might be caused by the fact that the fluctuations (thermal and quantum) responsible for the suppression of the long-range superconducting order became more important. 

Intrinsic superconductivity in the 1T MoS_2_ phase without electron injection was also investigated recently [133]. From the magnetic and electrical measurements, the superconductivity of 1T-MoS_2_ crystals was observed under 4 K, and the zero temperature upper critical magnetic field of 1T-MoS_2_ is estimated to be 5.02 T.

## 3. Preparation

Great efforts have recently been devoted to the preparation of low-dimensional TMDs, the details of which are as follows: 

### 3.1. Mechanical Cleavage Method

**Figure 5:**
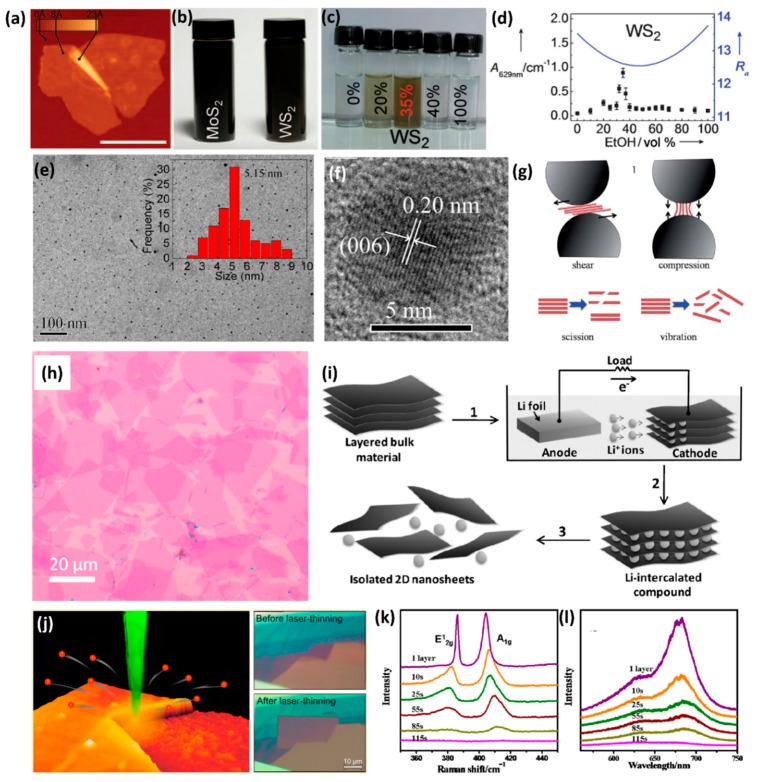
Top-down methods for the preparation of low-dimensional TMDs. (**a**) AFM image of as-exfoliated NbSe_2_. The scale bar is 1 μm. The different brightness corresponded to different thickness [30]. (**b**) Photographs of the dispersions of MoS_2_ and WS_2_ in NMP [32]. (**c**) Photographs of WS_2_ dispersions in various ethanol/water mixtures, which were stored for a week. The dispersion of WS_2_ reached its maximum concentration in 35 vol.% ethanol/water [33]. (**d**) The calculated R_a_ values (solid line) and the absorbance (dots) of the WS_2_ in various ethanol/water mixtures [33]. (**e**) Low-magnification TEM image of the MoS_2_ QDs. The size distribution of the QDs was shown in the inset, which indicated that the size was in the range of 2–9 nm [29]. (**f**) High-resolution TEM images of a typical MoS_2_ QDs. The clear lattice fringe indicated its highly crystalline structure [29]. (**g**) The illustration of shear and compression effects during the low-energy ball milling process, and the scission and vibration effects during the sonication process. The shear force induced exfoliation and the compression force induced exfoliation (**top**). Sonication induced scission and vibration induced exfoliation (**down**) [134]. (**h**) Optical image of the exfoliated monolayer TaS_2_ on SiO_2_/Si substrate. The size ranged from several tens of micrometers to more than 100 μm [135]. (**i**) Schematic drawing of the electrochemical lithiation process for the fabrication of 2D nanosheets from bulk material [36]. (**j**) **Left**: schematic illustration of the laser induced thinning method to prepare monolayer MoS_2_. **Right**: optical image of a multilayer MoS_2_ flake before and after scanned by a laser. The regions with different colors corresponded to different number of layers. After the laser-thinning process, the optical contrast of the rectangle region was uniform, consistent with that of a single MoS_2_ monolayer [37]. (**k**,**l**) Raman and PL spectra of monolayer MoS_2_ after Ar^+^ plasma irradiation with different time. On increasing the irradiation from 10 to 85 s, the Raman peaks became weak and broadened, and the PL intensities became much weaker. After 115 s irradiation, the Raman and PL peaks disappeared [38]. Reproduced with permission from [29]. Copyright AIP Publishing LLC, 2015. Reproduced with permission from [30]. Copyright Proceedings of the National Academy of Sciences of the United States of America, 2005. Reproduced with permission from [32]. Copyright American Association for the Advancement of Science, 2011. Reproduced with permission from [33,36]. Copyright Wiley-VCH Verlag GmbH & Co. KGaA, Weinheim, 2011. Reproduced with permission from [37]. Copyright American Chemical Society, 2012. Reproduced with permission from [38]. Copyright American Chemical Society, 2013. Reproduced with permission from [134]. Copyright The Royal Society of Chemistry, 2012. Reproduced with permission from [135]. Copyright American Chemical Society, 2017.

Mechanical cleavage method is a traditional way to obtain few-layer or monolayer TMDs [30,31]. In 2004, Novoselov’s group prepared graphene from highly-oriented pyrolytic graphite by using the mechanical cleavage method [1]. They also extended this method to other layered materials like BN, MoS_2_, NbSe_2_, Bi_2_Sr_2_CaCu_2_O_x_, and so on [30]. Just like drawing on a blackboard with chalk, the typical procedure of this method entails rubbing a fresh surface of a layered crystal against a clean substrate, like SiO_2_/Si substrate, leaving a variety of flakes attached to it. Examined under an optical microscope carefully, 2D samples with various layers including monolayer were observed, and the thickness of the as-exfoliated sample could be identified by optical contrast. To further determine the thickness, the Raman spectrometer, PL spectrometer, and AFM can also be used. The AFM image of as-exfoliated NbSe_2_ is shown in Figure 5a [30]; the varying thickness of the samples is portrayed by different colors. The bright color corresponds to the thick sample and the dark color corresponds to the thin one. The mechanical cleavage method can also be implemented with the aid of the Scotch-tape [27]. The typical procedure was as follows: The Scotch-tape was initially pressed onto the bulk crystal lightly and then removed carefully at a small angle. After this step, many thick sheets remained on the Scotch tape. The thin 2D nanosheets were obtained on repeatedly folding and peeling of the Scotch tape with thick sheets. The obtained 2D thin nanosheets were then transferred onto the pre-cleaned substrate by pressing the Scotch-tape with as-exfoliated nanosheets onto the SiO_2_/Si substrate. After a slight pressure with tweezers, the Scotch-tape was removed from the substrate slowly and as-exfoliated nanosheets with various thickness were left on the substrate.

Mechanical cleavage method is the most convenient method to prepare the 2D layered TMDs. However, the yield of the as-prepared sample is very low, which can just meet the needs of laboratory research and hinder large-scale applications. 

### 3.2. Liquid Exfoliation Method

Coleman et al. successfully exfoliated the layered materials by using the solvent-based method [32]. It was suggested that a successful solvent should meet two criteria. The first one is that the material must be suspended or dispersed in it for a reasonable amount of time. The second one is that the dispersed material must be highly exfoliated. Many common solvents have been experimented with in a bid to exfoliate the layered materials by sonication and centrifugation. The results indicated that surface tension of the solvents played a key role in the process. When the surface tension of the solvent matched with that of the layered material, it shows the best exfoliation and dispersity properties. For example, good dispersion of MoS_2_ and WS_2_ occurred for solvents with surface tensions in the range of 30–40 mJ/m^2^. Figure 5b shows that the dispersions of MoS_2_ (with the concentration of 0.3 mg/mL) and WS_2_ (with the concentration of 0.15 mg/mL) in NMP are stable over a period of hundreds of hours [32]. Besides NMP, commonly used solvents like water and ethanol can also be used to exfoliate the layered TMDs. Zhou et al. tried water, ethanol and ethanol/water mixtures as solvents to disperse MoS_2_ and WS_2_ [33]. Their findings indicate that MoS_2_ and WS_2_ could hardly be dispersed in pure ethanol or water, while ethanol/water mixtures with different compositions exhibited significantly different dispersion properties. The 35% volume fraction (35 vol.%) of ethanol in water, especially, represents the highest dispersion concentration of WS_2_ with 0.032 ± 0.003 mg/mL, and the suspensions could be stored for a week under ambient conditions (Figure 5c) [33]. In accordance with the theory of Hansen solubility parameters (HSP), the level of adaptation between the solvent and solute can be evaluated by the HSP distance R_a_. The smaller the R_a_ is, the better the solubility will be (Figure 5d) [33]. Theoretical calculations indicated that the 35 vol.% ethanol–water mixture had the minimum value of R_a_, which agreed well with the results observed in the experiment.

Gerchman et al. also tried an aqueous solution with surfactant as the solvent to exfoliate and suspend the WSe_2_ [136]. To prepare the exfoliation solvent, anionic surfactant sodium dodecyl sulfate (SDS) was mixed with deionized water at a concentration of 2 g/L. They found that the surfactant system resulted in the highest concentration after sonication and centrifugation as compared to the pure solvents and solvent mixtures, such as acetone, NMP, 60% ethanol in water and 30% propan-2-ol in water. The high concentration was mainly caused by the fact that the surfactant could create a bond with the newly exposed surface of the WSe_2_, so that each newly exfoliated layer could sterically and electrostatically be separated and stabilized. Moreover, the SDS solution could also stabilize the as-exfoliated suspension without noticeable precipitation after long periods of time. 

Furthermore, liquid exfoliation technique could also be used to prepare TMDs QDs [29,94,137,138,139]. Typically, commercial TMDs microcrystals are firstly dispersed in a solvent, such as mixture of ethanol and water, 1 methyl-2-pyrrolidone or DMF. After sonication (with bath sonication and probe sonication) and centrifugation, the supernatant with QDs was obtained. Figure 5e shows the TEM image of the MoS_2_ QDs [29]. Statistical size distribution indicated that the size was in the range of 2–9 nm and the most probable size is about 5.2 nm, as shown in Figure 5e [29]. The clear lattice fringe observed from the high-resolution TEM images implied that their highly crystalline structure was retained after the sonication treatment (Figure 5f) [29]. 

### 3.3. Low-Energy Ball Milling Assisted Method

The product concentration achieved by the liquid-phase exfoliation method as discussed in Section 3.2 is not enough for the scalable production of 2D nanosheets. In order to make the thinning process more efficient and scalable, Wong’s group reported a combined low-energy ball milling and sonication procedure for thinning and exfoliation of layered materials on a large scale [134]. Primarily, initial powder such as MoS_2_, was dispersed in 0.05 weight% surfactant sodium dodecyl sulfate (SDS)–water solutions. Then the dispersion was transferred to planetary mill and the low-energy ball milling process were carried out with rotation speed of 100 rpm. During the ball milling process, shear force and compression force were exerted on MoS_2_. The shear force could cleave the layers along the layer surface and the compression force could peel off thin MoS_2_ nanosheets from the edges as shown in Figure 5g [134]. After 12 h ball milling, the milled sample was taken out followed by centrifugation at 5000 rpm for 20 min to remove aggregates. Afterwards, 2 h sonication process with the power of 80 W was carried out to further exfoliate layered materials. The sonication process could induce scission, which can break larger crystallites into smaller crystallites. The sonication process could also induce vibration, which could chip off the thin 2D nanosheets from the outer surfaces [134,140]. A schematic diagram is shown in Figure 5g [134]. After low-energy ball milling and sonication, MoS_2_ nanosheets with concentration of 0.8 mg/mL were obtained. The diameters of the initial MoS_2_ ranged from 0.5 to 10 μm. After exfoliation, the size of MoS_2_ nanosheets was in the range of 50 nm to 700 nm and thickness was in the range of 1.2 nm to 8 nm.

### 3.4. Li-Intercalation Exfoliation Method

Joensen et al. developed a Li-intercalation exfoliation method to prepare a few-layer or monolayer MoS_2_ [35]. The typical procedure is as follows: Firstly, they soaked the 2H-MoS_2_ powder in a solution of n-butyl lithium in hexane (1.6 M), and kept it in this state for at least 48 h. In the process, lithium was intercalated into the interlayer of MoS_2_, forming Li*_x_*MoS_2_. The insertion of Li^+^ ions expanded the interlayer distance, which weakened the van der Waals interactions between the adjacent layers. After lithium intercalation, the Li_x_MoS_2_ was washed repeatedly with hexane, and then immersed in water. During the immersion process, the reaction between lithium and water generated H_2_ gas, which made the MoS_2_ layers go further apart from each other. With the aid of sonication, the final 2D MoS_2_ dispersion liquid was obtained [35]. 

Recently, with the modified method, Wu’s group prepared very-large-sized TMDs monolayers from fast exfoliation by manual shaking [135]. Instead of powder, they trialed the exfoliation with pristine MX_2_ (M = group IVB-VIB, X = S, Se) single crystals. The crystals mixed with 1.6 M n-BuLi in hexane was sealed in a quartz-lined autoclave and kept at 100 °C. By deliberately controlling the reaction time, Li*_x_*MX_2_ with different lithium contents was obtained and gigantic expansion (∼94%) in Li*_x_*MX_2_ single crystals was realized. The gigantic expansion enabled exfoliation with a gentle-driving force. By only manual shaking within several seconds, expanded Li*_x_*MX_2_ crystals were exfoliated into homogenetic monolayers of submillimeter scale sizes and high crystallinity. Figure 5h represents the characteristic optical images of the exfoliated TaS_2_ monolayers with sizes ranging from several tens of micrometers to more than 100 μm [135]. DFT calculations indicated that the insertion of lithium into MX_2_ would induce lattice distortions along the in-plane direction and change the lattice parameter. Study on 13 kinds of TMDs materials demonstrated that the lateral size of the as-exfoliated monolayer was largely determined by the lattice strain in the Li*_x_*MX_2_ crystals. The large compressive strains in the lithium-intercalated compounds led to a small-size monolayer sample.

Zhang’s group developed an electrochemical Li-intercalation exfoliation method to prepare the few-layer or monolayer TMDs [36]. Figure 5i gives a schematic illustration of the electrochemical Li-intercalation process in a battery test system, in which the layered bulk material (MoS_2_, WS_2_, TiS_2_, TaS_2_, etc.) was used as the cathode, and the Li foil as anode [36]. During the discharge process, the lithium was intercalated into the interlayer of the layered bulk material. On completion of lithium insertion, the intercalated compound was sonicated in water or ethanol to exfoliate the 2D nanosheets. 

### 3.5. Laser and Plasma-Induced Thinning Method

To exploit thickness-dependent properties, a decisive factor is to control the thickness of the TMDs. Steele’s group developed a technique to controllably thin out the multilayered MoS_2_ down to a monolayer by using laser [37]. A schematic diagram is shown in Figure 5j [37]. During the laser-thinning progress, the laser scanned point-by-point along the surface of the MoS_2_ flakes. The dose of the laser could be determined by adjusting step size, exposure time, and the power of incident laser. After several trials, they found that a 400-nm step size with 0.1 s exposure time and 10 mW of incident power could effectively thin out MoS_2_ flakes with thickness of 20 layers. Figure 5j shows the optical image of MoS_2_ flake on SiO_2_/Si substrate, before and after the laser-thinning respectively. After the laser-thinning process, the laser scanning area showed uniform optical contrast. AFM characterization indicated that the thickness of the scanning area was 0.9 ± 0.3 nm, consistent with the thickness of monolayer MoS_2_. Compared with monolayer MoS_2_ prepared by mechanical cleavage method, the as-thinned monolayer sample showed similar semiconducting properties, which were confirmed by the PL, Raman vibration and electronic transport characterizations. 

Ni’s group demonstrated that Ar^+^ plasma irradiation was also an effective way to regulate the thickness of MoS_2_ [38]. The power and pressure of Ar^+^ plasma were chosen as 50 W and 40 Pa, respectively. By increasing the irradiation time from 10 s to 85 s, the Raman and PL peaks of monolayer MoS_2_ became weaker and broader, which indicated that the structure of MoS_2_ became disordered. When the irradiation time was up to 115 s, the Raman and PL peaks disappeared, which demonstrated that MoS_2_ layer was totally removed (Figure 5k,l) [38]. At the same time, they also conducted Ar^+^ plasma irradiation on bilayer MoS_2_. When the irradiation time was increased from 0 s to 115 s, the $E_{2g}^{1}$ and *A*_1*g*_ peaks gradually shifted to the frequency of the monolayer MoS_2_. As for the PL spectra, the PL intensity had an abrupt increase after 115 s irradiation, which indicated that the top layer has been totally removed, and the MoS_2_ has transformed from bilayer to monolayer. 

### 3.6. Thermal Annealing Method

Besides the laser and plasma-induced thinning method, thermal annealing in a controlled atmosphere can also be used to thin TMDs [141,142]. Taking MoS_2_ as an example, the typical procedure is as follows: At first, scotch tape was used to exfoliate few-layer MoS_2_ flakes from its bulk form and the as-exfoliated flakes were transferred to SiO_2_/Si substrate subsequently. To conduct the thermal annealing process, the samples with silicon substrate were placed at the center of the CVD quartz tube. During annealing temperature, pressure and argon gas flow rate were set to 650 °C, 10 Torr and 5 sccm, respectively. It was discovered that one layer was lost after one hour of annealing. By increasing the annealing time, few-layer MoS_2_ flakes could be peeled off layer by layer, which was confirmed by microspectrophotometry, Raman spectroscopy and AFM characterization. At the same time, the surface area showed a shrinkage during the annealing process, which indicated that sublimation occurred perpendicularly on the surface and along the surface, simultaneously. However, in repeated experiments, most atomically thin MoS_2_ flakes appeared as inhomogeneous surfaces, which might be caused by vacancy defects. During annealing, vacancies could form randomly in any area because of sublimation and the vacancies could make their surrounding parts easier to sublimate due to broken covalent bonds.

### 3.7. Solid-Phase Reaction Method

**Figure 6:**
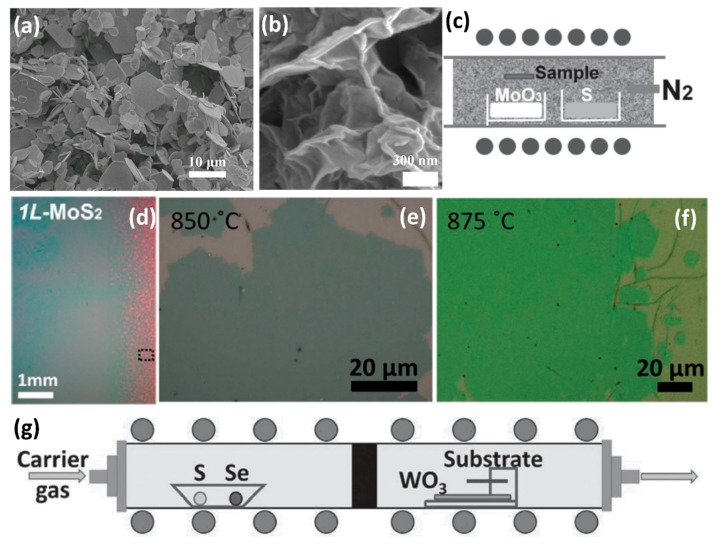
Bottom-up approaches (solid-phase reaction method, hydrothermal method and CVD method) for the preparation of 2D TMDs. (**a**) SEM image of MoS_2_ flakes synthesized by solid-phase reaction method. The lateral size of MoS_2_ flakes could be up to 10 μm, and the thickness was about a few hundred nanometers [143]. (**b**) SEM image of MoS_2_ nanosheets synthesized by the hydrothermal method. The 2D MoS_2_ nanosheets were rolled up and formed a nanoflower morphology [39]. (**c**) Schematic illustration of the setup for the synthesis of monolayer MoS_2_. The substrates were placed face-down above the ceramic boat, which was filled with MoO_3_. Another ceramic boat filled with S powder was located in the upstream of MoO_3_ [41]. (**d**) Optical image of monolayer MoS_2_. The MoS_2_ showed a uniform morphology with centimeter sizes [42]. (**e**,**f**) Optical images of monolayer WSe_2_ synthesized at different growth temperatures by CVD method. The monolayer WSe_2_ exhibited the uniform morphologies with several tens microns in size [43]. (**g**) Schematic depiction of the growth setup for the synthesis of monolayer WS_2(1−*x*)_Se_2*x*_ alloys. The growth was conducted in a two-temperature zone tube furnace, where S and Se powders were placed in the upstream zone, and the WO_3_ was placed in the downstream zone [108].Reproduced with permission from [39]. Copyright Macmillan Publishers Limited, 2014. Reproduced with permission from [41]. Copyright Wiley-VCH Verlag GmbH & Co. KGaA, Weinheim, 2012. Reproduced with permission from [42]. Copyright American Chemical Society, 2013. Reproduced with permission from [43]. Copyright The Royal Society of Chemistry, 2015. Reproduced with permission from [108]. Copyright Wiley-VCH Verlag GmbH & Co. KGaA, Weinheim, 2015. Reproduced with permission from [143]. Copyright Elsevier B.V., 2017.

Bottom-up chemical methods offer potentially powerful alternative exfoliation routes for preparing TMD nanosheets. Among the various ways to prepare TMDs, the solid-phase reaction method provides easier operation with good repeatability. During the synthesis, the transition metal and chalcogen powders were chosen as the precursors [143]. For the synthesis of 2D MoS_2_ flakes, the molybdenum powder and sulphur powder were mixed in a stoichiometric ratio and loaded in a pre-cleaned quartz tube. After purging the quartz tube with ultrahigh-purity argon, the quartz tube with precursors was sealed. Then the sealed quartz tube was heated to 1000 °C at a rate of 15 °C/min and kept at 1000 °C for 3 days. After cooling to room temperature naturally, 2D MoS_2_ flakes were obtained. The typical SEM image of the as-synthesized MoS_2_ flakes is shown in Figure 6a. The lateral size of MoS_2_ flakes could be up to 10 μm, and the thickness was about a few hundred nanometers. At the same time, by mixing the molybdenum (tungsten), sulphur and selenium powders in a stoichiometric ratio, MS_2(1−*x*)_Se_2*x*_ (M = Mo, W) alloys with different ratio of S and Se were achieved [143]. 

### 3.8. Hydrothermal Method

Hydrothermal method is also an effective way to synthesis 2D TMD nanosheets. Our group synthesized MoS_2_ nanosheets by using MoO_3_ and KSCN as the reactants [39]. MoO_3_ and KSCN with the molar ratio of 1:3 were dissolved in 80 mL distilled water. Subsequently, 0.28 mL HCl at a concentration of 12.5 mol/L was added into the reaction solution under violent stirring. Then the as-prepared solution was transferred into a 100 mL Teflon-lined stainless autoclave. After sealing tightly, the autoclave was heated in the oven at 240 °C for 24 h. The autoclave was then allowed to cool down at room temperature naturally. The reaction product was filtered off, washed with distilled water and absolute ethanol, and then dried in vacuum at 60 °C for 6 h. The morphology of the as-synthesized MoS_2_ nanosheets was characterized by SEM, which showed that the 2D MoS_2_ nanosheets rolled up, thus forming a nanoflower morphology (Figure 6b) [39]. 

Besides the MoO_3_ and KSCN, (NH_4_)_2_MoS_4_ could also be a precursor for the synthesis of MoS_2_ nanosheets via hydrothermal method [144]. By adjusting the growth temperature and time at 220 °C and 12 h, respectively, Wang et al. also synthesized the MoS_2_ nanosheets with different diameters. They also integrated 2D MoS_2_ nanosheets with drug delivering implant for highly-efficient near infra-red triggered synergistic tumor hyperthermia. The details can be found in Section 4.5. 

At the same time, hydrothermal method can also be used to prepare TMDs QDs [40,145,146,147]. Wang et al. synthesized MoS_2_ QDs by using Na_2_MoO_4_·2H_2_O and L-cysteine as the precursors [147]. In brief, the Na_2_MoO_4_·2H_2_O was primarily dissolved in water, and then the solution was adjusted to pH 6.5 with HCl. Afterwards, the l-cysteine and water were added to the above solution followed by the ultrasonication process. Then the mixture was transferred into autoclave and reacted at 200 °C for 36 h. After centrifugation, the supernatant containing MoS_2_ QDs were obtained. Furthermore, the hydrothermal process is also beneficial to the cutting of the basal plane when preparing TMDs QDs. Taking MoS_2_ QDs as an example, the short time ultrasound treatment of MoS_2_ nanosheets with subsequent hydrothermal process produced MoS_2_ QDs with a size distribution of ~2.0 ± 0.5 nm in diameter [141].

### 3.9. CVD Method

The lateral size of the 2D TMDs synthesized by the aforementioned methods is very small, an order of several micrometers, which hampers industrial applications in the future. Therefore, the methods for preparing 2D TMDs by the CVD method are of wide concern. In general, when the CVD method is adopted to synthesize few-layer or monolayer TMDs, the products are always grown on SiO_2_/Si substrates, sapphire, Au foils or quartz substrates by using transition metal oxide (MO_3_) and chalcogen (X) powders as precursors [41,42,43,44,148,149,150]. For example, monolayer MoS_2_ was grown on SiO_2_/Si substrate in a quartz tube furnace at atmospheric pressure by using N_2_ or Ar gas as the carrier gas. Figure 6c shows a schematic illustration of the experimental setup [41]. The S powder was located at the upstream of the tube, while the MoO_3_ powder with the substrate placed face-down above was placed at the downstream of the quartz tube. Prior to growth, the SiO_2_/Si substrates were treated by graphene-like molecules, such as reduced graphene oxide (rGO), perylene-3,4,9,10-tetracarboxylic acid tetrapotassium salt (PTAS) and perylene-3,4,9,10-tetracarboxylic dianhydride (PTCDA), which could promote surface nucleation efficiency and layer growth [41,42]. During the growth process, the MoO_3_ powder was firstly reduced by sulfur vapor to form volatile sub-oxide MoO_3−*x*_ [35,36]. Then the MoO_3−*x*_ molecule was further reacted with sulfur vapor to form monolayer MoS_2_ on the substrates [35,36]. Figure 6d shows the optical image of the uniform MoS_2_ monolayers with several centimeters in size, which are grown on a SiO_2_/Si substrate [42]. By using varying growth parameters, the transition between vertical and horizontal growth was achieved [151,152].

The chemical reactivity of Se is much lower than that of S [107,153]. As a consequence, compared with transition metal disulfides, transitional metal diselenium compounds like WSe_2_ and MoSe_2_ were difficult to synthesize. Our group successfully synthesized the monolayer WSe_2_ by using a mixture of argon–hydrogen (5%) gas as the carrier and reduction gas [43]. With the introduction of H_2_, the reduction of WO_3_ to WO_3−*x*_ became much easier, facilitating subsequent reactions. During the synthesis process, we initially heated the furnace to 925 °C, then conducted a sub-cooling step to a lower temperature and maintained the lower temperature for 15 min. This lower temperature was very important for the growth of monolayer WSe_2_. Figure 6e,f show the optical images of as-synthesized WSe_2_ at different growth temperatures, which exhibit uniform morphologies with several tens microns in size [43]. AFM, Raman and PL characterizations confirmed the monolayer configuration in the as-grown WSe_2_. By optimizing the synthesis conditions, we also synthesized the monolayer WS_2(1−*x*)_Se_2*x*_ alloys with different Se contents [108,109]. Figure 6g shows a schematic illustration of the growth setup. In the upstream of the furnace, the ceramic boat loaded two reactants, the S powder and Se powder. Meanwhile, the WO_3_ powder was evenly spread on a quartz holder located in the downstream. We also performed atomic-scale analysis of the WS_2(1−*x*)_Se_2*x*_ alloys by using the HRSTEM, details of which are provided in Section 2.1. 

During the growth of TMDs by CVD method, mass flux and growth rate played a key role in the morphology of the TMDs, where mass flux determined the amount of metal precursors involved in the formation of the nucleus and the growth of domains, and the growth rate dominated the grain size [154]. Many transition metals or metal oxides have high melting points and low vapor pressure, which lead to very low mass flux and limited reaction [154]. Therefore, many of the layered TMDs are difficult to produce via the CVD method. Inspired by the fact that the molten-salt could be used to produce ceramic powders at relatively low temperature, Zhou et al. demonstrated that molten-salt-assisted CVD could be broadly applied for the synthesis of a wide variety of TMDs in atomically thin, including 32 binary compounds, 13 alloys and two heterostructured compounds [154]. Thermogravimetry and differential scanning calorimetry measurements indicated that the melting points of salts mixed with the metal sources fell in the range of 600 °C to 850 °C. The molten salts reduced the melting points of the metal precursors or formed oxychlorides via reaction with some metal oxides so as to increase the mass flux and the rate of the reaction. The universal method for the synthesis of 2D TMDs facilitated the exploration of their physical properties and applications. 

For real applications, the controlled growth of large-area uniform monolayer TMDs on low cost substrates is very important. Very recently, Zhang’s group realized the direct synthesis of a 6-inch uniform monolayer MoS_2_ on solid soda-lime glass through a face-to-face metal-precursor supply route in a facile CVD process [155]. Experimental study demonstrated that the continuous 6-inch monolayer MoS_2_ film was synthesized at the edge growth rate of around 1.2 μm/s. This growth rate was much faster than that on common insulating substrates, such as SiO_2_/Si (0.4 μm/s) and sapphire (0.2 μm/s). This high growth efficiency was proven to be facilitated by uniformly distributed Na in soda-lime glass, which served as perfect catalysts for rapid and large-scale uniform growth. DFT calculations indicated that Na adsorption could reduce the energy barriers for MoS_2_ growth along the S-terminated edges and Mo-terminated edges, which agreed well with the experimental results.

**Figure 7:**
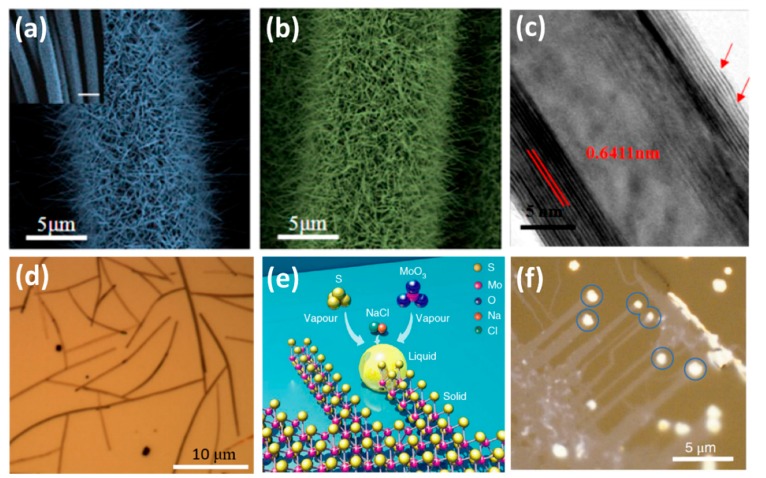
CVD synthesis of 1D TMDs. (**a**,**b**) SEM images of WO_3_ nanowires and WS_2(1−*x*)_Se_2*x*_ nanotubes, respectively. The entire surfaces of the CFs were covered vertically and uniformly with high-density WO_3_ nanowires and WS_2(1−*x*)_Se_2*x*_ nanotubes. The WO_3_ nanowires showed the length of ∼5 μm and the diameter of ∼100 nm [45]. (**c**) HRTEM image of WS_2(1−*x*)_Se_2*x*_ nanotubes. The spacing of WS_2(1−*x*)_Se_2*x*_ nanotubes between lattice fringes was 0.6411 nm [45]. (**d**) Optical image of MoS_2_ nanobelts. The area coverage was around 5%, and the length was 10~20 μm [80]. (**e**) Schematic illustration of the growth of MoS_2_ narrow ribbons. Firstly, the liquid phase Na–Mo–O in small droplets was formed. Then the Na–Mo–O droplet dissolved sulfur. At last, the ribbons grew horizontally and the droplet crawled laterally [50]. (**f**) Optical image of MoS_2_ ribbons grown on a NaCl crystal. The ribbons showed the widths of a few tens of nanometers to a few micrometers and lengths ranged from a few to tens of micrometers [50]. Reproduced with permission from [45]. Copyright American Chemical Society, 2014. Reproduced with permission from [50]. Copyright Macmillan Publishers Limited, 2018. Reproduced with permission from [80]. Copyright American Chemical Society, 2015.

Xu et al. also successfully synthesized the one-dimensional WS_2(1−*x*)_Se_2*x*_ nanotubes via the selenization and sulfurization process [45]. The carbon fibers (CFs) with wonderful electric conductivity, high thermal and chemical stabilities were chosen as the substrate in their synthesis. Firstly, WO_3_ nanowires were synthesized by a catalyst-free physical vapor deposition method. The WO_3_ powder, which evaporated at high temperature deposited on the CFs, showed the nanowires morphology at a vacuum lower than 1 Pa at inert atmosphere. CFs with large coverage of WO_3_ nanowires were then placed in a tube furnace and S and Se powders were chosen as the precursors to transfer the WO_3_ nanowires into WS_2(1−*x*)_Se_2*x*_ nanotubes. Figure 7a,b show the typical SEM images of the WO_3_ nanowires and WS_2(1−*x*)_Se_2*x*_ nanotubes, respectively. The entire surfaces of the CFs were vertically and uniformly covered with high-density WS_2(1−*x*)_Se_2*x*_ nanotubes. The HRTEM characterization (Figure 7c) indicated that the as-synthesized one-dimensional WS_2(1−*x*)_Se_2*x*_ sample, showing multiwall nanotubes morphology. CVD method can also be used to prepare the 1D MoS_2_ nanobelts [49,80]. Recently, our group successfully synthesized one-dimensional MoS_2_ nanobelts by using n-type silicon as the substrate [80]. Figure 7d shows the optical image of the MoS_2_ nanobelts. The length was about 10–20 μm, and the area coverage was around 5%. As discussed in Section 2.1, the (001) basal planes of the MoS_2_ nanobelt were standing vertically on the substrate and the edges of the base planes formed the top surfaces of the nanobelt [90]. We also realized the control of its chemical and physical properties by forming MoS_2(1−*x*)_Se_2*x*_ nanobelts alloys through optimization of growth conditions [156].

With the aid of alkali metal halide, the highly crystalline monolayer MoS_2_ ribbons with a width of few tens to thousands of nanometers were also successfully synthesized [50]. During this growth, the MoO_3_ reacted with NaCl and formed the molten Na–Mo–O droplets. These droplets crawled on the substrate surface and mediated the highly anisotropic growth of MoS_2_ ribbons when saturated with sulfur. With the lateral displacement of the droplet, the horizontal growth of ribbons kept going. The schematic illustration is shown in Figure 7e [50]. Figure 7f shows the optical image of MoS_2_ ribbons grown on a NaCl crystal substrate [50]. Differing from the typical triangular morphology, ribbons with lengths from a few to tens of micrometers, and widths of a few tens of nanometers to a few micrometers were grown. The bright dots indicated by the circles were characteristic features of nanostructures that resulted from the crawling mode.

### 3.10. Annealing of the (NH_4_)_2_MoS_4_ Precursor

**Figure 8:**
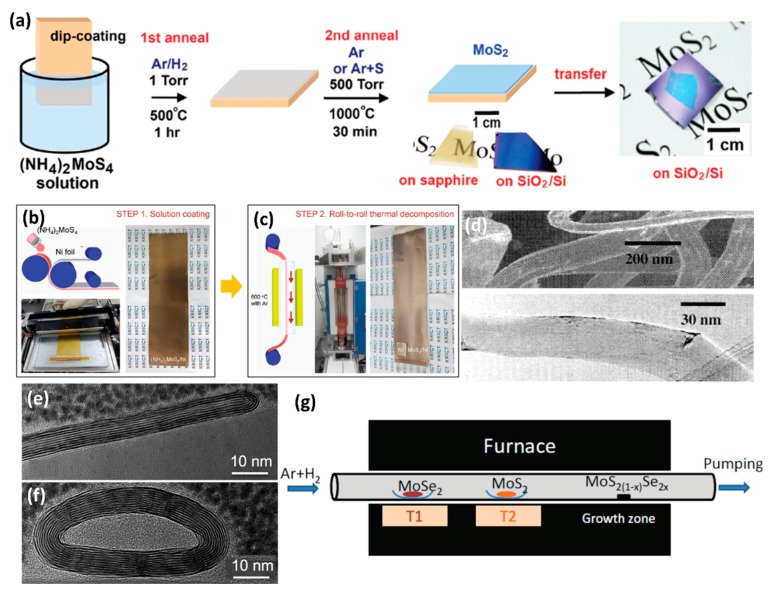
Bottom-up approaches (annealing of the (NH_4_)_2_MoS_4_ precursor, chemical vapor transport method and physical vapor deposition method) for the preparation of low-dimensional TMDs. (**a**) Schematic depiction of the two-step thermolysis process for the synthesis of MoS_2_ thin layers. The insulating substrate was immersed into the (NH_4_)_2_MoS_4_ solution followed by the two-step annealing process [51]. (**b**,**c**) Photographs and schematic illustration of coating of (NH_4_)_2_MoS_4_ on Ni foils and roll-to-roll thermal decomposition for layer-controlled MoS_2_ on Ni foils [157]. (**d**) SEM (**top**) and TEM (**down**) images of MoS_2_ nanotubes. The MoS_2_ nanotube had a typical length of several hundreds of nanometers and a uniform diameter about 50 nm [158]. (**e**) Typical TEM image of MoS_2_ nanoribbon. At the edge of the nanoribbon, it showed the wrapping of the layers [54]. (**f**) Cross-section TEM image of MoS_2_ nanotubes. The nanotubes had minor and major radii of 5 nm and 20 nm, respectively [54]. (**g**) Schematic illustration of three-zone furnace for the physical vapor deposition growth of monolayer MoS_2(1−*x*)_Se_2*x*_. MoSe_2_ and MoS_2_ powders were put in the first and second upstream zones, respectively, and the substrate was put in the third zone. During the growth, the temperature of the third zone was ∼600–700 °C, which was higher than that of the first and second zones (940–975 °C) [48]. Reproduced with permission from [48]. Copyright Wiley-VCH Verlag GmbH & Co. KGaA, Weinheim, 2014. Reproduced with permission from [51]. Copyright American Chemical Society, 2012. Reproduced with permission from [54]. Copyright AIP Publishing LLC, 2015. Reproduced with permission from [157]. Copyright Wiley-VCH Verlag GmbH & Co. KGaA, Weinheim, 2017. Reproduced with permission from [158]. Copyright American Chemical Society, 2001.

Besides the transition metal oxide (MO_3_), other compounds like ammonium thiomolybdate ((NH_4_)_2_MoS_4_) can also be reactants during the synthesis of few-layer TMDs. Liu et al. reported the successful synthesis of large area MoS_2_ thin sheets on a variety of insulating substrates by the thermal decomposition of (NH_4_)_2_MoS_4_ in the presence of sulfur vapor [51]. The schematic illustration is shown in Figure 8a [51]. Firstly, the (NH_4_)_2_MoS_4_ was dip-coated on SiO_2_/Si or sapphire substrates. Then, a two-step annealing processes were carried out to transfer the (NH_4_)_2_MoS_4_ into MoS_2_ films. The first annealing process was carried out at a low pressure (1 Torr) in an Ar/H_2_ atmosphere (flow rate 4:1) to efficiently remove the residual solvent, NH_3_ molecules, and other byproducts dissociated from the precursors. Compared with the annealing temperature of 500 °C in the first annealing process, the high-temperature annealing at 1000 °C in Ar or Ar + S atmosphere was used to improve the MoS_2_ crystal structure. The electrical properties of transistor devices fabricated with as-prepared MoS_2_ thin layers were comparable with that of mechanically exfoliated MoS_2_.

To trigger actual industrial applications, Lim et al. developed a facile method for the large-scale production of MoS_2_ layers by using the roll-to-roll manufacturing technology [157]. During the synthesis, they chose inexpensive Ni foils as the substrate and achieved 50 cm long MoS_2_ layers with excellent long-range uniformity and optimum stoichiometry. Initially, a (NH_4_)_2_MoS_4_ solution in ethylene glycol was bar-coated onto a hydrophilic-treated 25 μm thick Ni foils by UV irradiation. The Ni foils were then placed in the roll-to-roll chamber and annealed with the introduction of N_2_ under a certain vacuum. Figure 8b,c present the schematic illustration and photographs of simple coating of (NH_4_)_2_MoS_4_ on Ni foils and roll-to-roll thermal decomposition of MoS_2_ [157]. At the same time, they established the optimized conditions for the synthesis of layer-controlled MoS_2_ by the precise control of the (NH_4_)_2_MoS_4_ concentration. To evaluate the layer homogeneity of such large area MoS_2_ samples, they performed Raman characterization at every 1 cm. Two typical Raman active modes ($E_{2g}^{1}$ and *A*_1*g*_) are observed over the entire area from all samples, which demonstrates that the synthesis yielded homogeneous MoS_2_.

Annealing of (NH_4_)_2_MoS_4_ with subsequent hydrogen treatment could effectively produce MoS_2_ and WS_2_ nanotubes [52,158]. For the preparation of MoS_2_ nanotubes, (NH_4_)_2_MoS_4_ was heated at 400 °C in an argon atmosphere, after which MoS_3_ was obtained [52]. The reaction involved was:

(NH_4_)_2_MoS_4_ → MoS_3_ + H_2_S + NH_3_

Heating the obtained amorphous MoS_3_ in a stream of H_2_ at 1200–1300 °C realized an excellent yield of nanotubes. To obtain WS_2_ nanotubes, similar reactions could be carried out with ammonium thiotungstate as the precursor [52].

Chen et al. also successfully synthesized MoS_2_ nanotubes by direct reaction of (NH_4_)_2_MoS_4_ and hydrogen at a relatively low temperature [158]. At first, (NH_4_)_2_MoS_4_ was pretreated by ball-milling under a hydrogen atmosphere for 1 h to “activate” the fine powders. Then, the active powder was sintered in a floating hydrogen/thiophene at a relatively low temperature of 400 °C. After 1 h growth, MoS_2_ nanotubes with specific surface area of 58 m^2^/g were obtained. As shown in Figure 8d, the as-synthesized MoS_2_ nanotube had a typical length of several hundreds of nanometers and a uniform diameter of about 50 nm [158].

### 3.11. Chemical Vapor Transport Method

Chemical vapor transport method is a traditional technique for the preparation of nonvolatile solids and has been used to produce single crystals of TMDs. For the growth of TMDs crystals, the precursors and transport agents (iodine) were sealed in a quartz ampoule under high vacuum. The sealed ampoule was then heated under a temperature gradient in which the I_2_ molecules could carry TMDs into the gas phase in the high temperature zone and release them at the cold end. To realize the direct growth of 2D TMDs, Hu et al. designed the experiments by modifying the reaction ampoule and tuning the growth parameters [53]. In the case of growing monolayer MoS_2_, the MoO_3_ and S were selected as precursors, and I_2_ was selected as a transporting agent. By reducing the amounts of MoO_3_ with S to about 1:1000–1:10,000 and I_2_ to 1:10, dramatical decrease of the growth rate was achieved, and the monolayer MoS_2_ was produced in fluorophlogopite mica at a growth temperature of 300–600 °C for 0.5–2 h. For example, with chemical vapor transport growth of 30–40 min at 400 °C, monolayer MoS_2_ triangular flakes with a side length of 30–60 mm were obtained. Besides MoS_2_, this method was also used in the growth of 2D WS_2_, MoSe_2_, Mo*_x_*W_(1−*x*)_S_2_ alloys and ReS_2_.

The chemical vapor transport could also be used to prepare TMDs nanotubes and nanoribbons with very slow rate [54,159]. For the preparation of MoS_2_ nanotubes or nanoribbons, MoS_2_ powder and iodine were sealed in silica ampoule at a pressure of 7 × 10^−4^ Pa. The iodine transported the evacuated MoS_2_ vapor phase with the temperature from 1133 K to 1010 K with a temperature gradient of 6.2 K/cm in a two-zone furnace. After three weeks of growth, the silica ampoules were cooled to room temperature with a controlled cooling rate of 60 °C/h, and a few percent of the starting MoS_2_ powder was transported by the reaction to form nanotubes. With continuous growth, some of the nanotubes would spontaneously collapse and form the ribbon shapes. The length of the nanotubes could be up to several millimeters in length, and the diameters in multiwall nanotubes ranged from several micrometers to less than ten nanometers. The typical TEM image of MoS_2_ nanoribbon and cross-section TEM image of MoS_2_ nanotubes is shown in Figure 8e,f [54]. At the nanoribbon edge, the layers have shown wrapping morphology, and the MoS_2_ nanotubes with a layer thickness of 11 monolayers had minor and major radii of 5 nm and 20 nm, respectively. 

### 3.12. Physical Vapor Deposition Method

Physical vapor deposition method is another efficient way to prepare 2D TMDs and their alloys [48,160,161]. Typically, the TMDs powder source was placed in the upstream of a horizontal quartz tube furnace and the cleaned substrate was placed downstream, far from the TMDs powder with a temperature gradient. By finely tuning the growth conditions, such as gradient of deposition temperature, evaporation temperature, gas flow, high-quality monolayer or few-layer TMDs were grown on substrate. For the growth of TMDs alloys, the difference was that two or more kinds of powder were placed in the upstream of a horizontal quartz tube furnace. By adjusting the molar ratio of the powder, different contents of transition metal or chalcogens were achieved in the 2D alloys. Figure 8g illustrated the setup used in the growth of the MoS_2(1−*x*)_Se_2*x*_ monolayer; MoSe_2_ and MoS_2_ powders were put in the first and second zones of the three-zone furnace, respectively.

## 4. Applications

### 4.1. Electronic Devices

According to Moore’s Law, the increase of transistors integration in semiconductor chips will result in the channel length of the transistors being below 10 nm [162]. To maintain good gate control of the channel and to minimize leakage current, channel thickness below 2 nm is required [163,164,165]. This is extremely challenging for silicon because ultrathin silicon suffers from surface roughness effects. The surface roughness scattering will reduce its mobility by nearly two orders of magnitude [163,164,165]. Fortunately, the emergence of novel classes, such as 2D layered TMDs, which do not have surface roughness, has opened up new ways in device design [166]. In 2005, Geim’s group initially investigated the electrical conductivity of mechanically cleaved 2D MoS_2_ [30]. The measured mobilities ranged from 0.5 to 3 cm^2^·V^−1^·s^−1^, which agreed well with the value achieved in its bulk form. The low mobility of MoS_2_ hampered its application in practical devices, which facilitated researchers to make great effects to improve its electrical performance. Jena et al. simulated the effect of dielectric engineering on the influence of carrier mobility in 2D and 1D semiconductor nanostructures [167]. Their results indicated that the scattering from Columbic impurities could be strongly damped when the nanostructures were coated with high-κ dielectrics. The damping of Columbic scattering improved the mobility of carriers, which made the nanostructures show better electrical behavior. 

**Figure 9:**
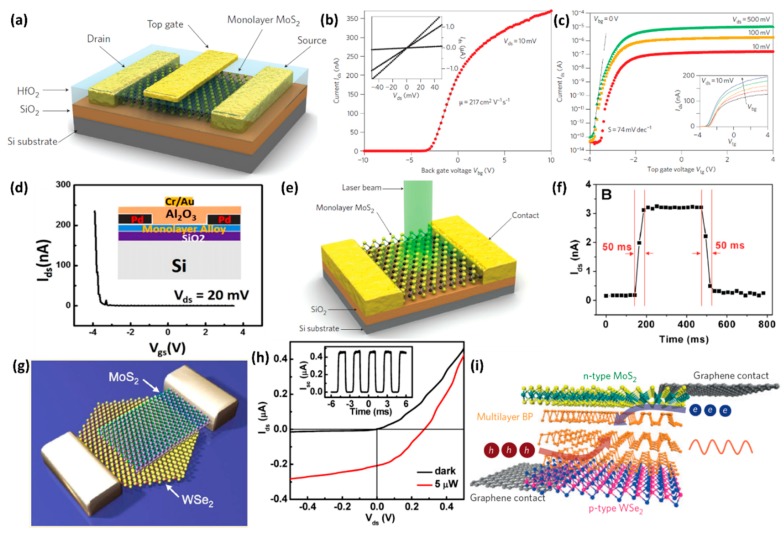
Application of low-dimensional TMDs in electronic devices and optoelectronic devices. (**a**) Schematic view of monolayer MoS_2_ based transistors. 30 nm HfO_2_ was chosen as the top gate dielectric because of its high dielectric constant of 25, band gap of 5.7 eV [31]. (**b**) Transfer characteristic of monolayer MoS_2_ FET with the bias voltage V_ds_ of 10 mV, from which the channel mobility of ~217 cm^2^·V^−1^·s^−1^ was estimated. The inset showed the I_ds_–V_ds_ curve acquired with V_bg_ values of 0, 1 and 5 V, which indicated that gold contacts were ohmic [31]. (**c**) I_ds_–V_tg_ curves of the MoS_2_ transistor with the bias voltage ranging from 10 mV to 500 mV. The measured current on/off ratio was higher than 1 × 10^8^, and subthreshold slope for the transition between the on and off states was 74 mV/dec [31]. (**d**) Transfer characteristic of top-gated monolayer WSe_2(1−*x*)_S_2*x*_ (*x* = 0.28) device with the bias voltage V_ds_ of 10 mV. The insert shows the schematic view of the transistors with the top-gate electrode [43]. (**e**) Schematic view of the single-layer MoS_2_ photodetector. During the measurement, the laser beam directly focused on the surface of MoS_2_ [168]. (**f**) Photoswitching rate of single-layer MoS_2_ phototransistor at V_ds_ = 1 V, P_light_ = 80 μW. The switching duration for the current rise or decay process was only ~50 ms [169]. (**g**) Schematic illustration of the WSe_2_/MoS_2_ vertical heterojunction device [170]. (**h**) The output curve of the WSe_2_/MoS_2_ heterojunction p-n diode with and without illumination. The output characteristics showed clear photovoltaic effect with an open-circuit voltage of ∼0.27 V and a short-circuit current of ∼0.22 μA. The inset showed temporal response of the photocurrent generation under 514 nm illumination (10 μW), from which the EQE was determined as the 11% [170]. (**i**) Schematic illustration of a 2D heterostructure LED. A multilayer BP thin film was sandwiched between the n-type MoS_2_ and p-type WSe_2_ which were used to inject the electrons and holes, respectively [171]. Reproduced with permission from [31]. Copyright Macmillan Publishers Limited, 2011. Reproduced with permission from [43]. Copyright IOP Publishing Ltd., 2016. Reproduced with permission from [168]. Copyright Macmillan Publishers Limited, 2013. Reproduced with permission from [169]. Copyright American Chemical Society, 2011. Reproduced with permission from [170]. Copyright American Chemical Society, 2014. Reproduced with permission from [171]. Copyright Macmillan Publishers Limited, 2014.

Inspired by their theoretical predictions, Kis’s group chose HfO_2_ as the high-κ dielectric and deposited it on the top surface of monolayer MoS_2_ by an atomic layer deposition method [31]. Followed by the electron-beam lithography process, the monolayer MoS_2_-based top-gated configuration device with HfO_2_ as the gate dielectric was fabricated, as shown in Figure 9a [31]. The width of the top gate was 4 μm and the top gate length, source–gate and gate–drain spacing was 500 nm. Figure 9b shows the room-temperature transfer characteristic for the device with the bias voltage of 10 mV, from which the mobility of ~217 cm^2^·V^−1^·s^−1^ was extracted. The I_ds_–V_tg_ curves with the bias voltage ranging from 10 mV to 500 mV in Figure 9c indicated that the room-temperature on/off ratio exceeded 10^8^ and the subthreshold slope for the transition between the on and off states was 74 mV/dec for a bias V_ds_ of 500 mV. These values were comparable to the performance of thin silicon films. However, for the back-gated configuration, the monolayer devices displayed mobility of 0.1–10 cm^2^·V^−1^·s^−1^, much lower than the previously reported phonon-scattering-limited room-temperature mobility. 

Lee et al. investigated the electrical properties of CVD growth TMDs [42]. Their results indicated that the on−off current ratio exceeds 10^7^; the room temperature and low temperature mobilities were up to 1.2 cm^2^·V^−1^·s^−1^ and 500 cm^2^·V^−1^·s^−1^ respectively. These values were comparable to the exfoliated monolayer MoS_2_ fabricated without high κ-dielectrics screening. Our group also studied the electrical performance of as-grown monolayer WSe_2_: the back gated configuration field-effect mobility was ∼0.2 cm^2^·V^−1^·s^−1^ and the hole carrier concentration was ∼1.11 × 10^18^ cm^−3^ [43]. This low field-effect mobility might be caused by the grain boundaries in the as-grown sample, which could scatter the carriers during transport. By partially substituting the Se atoms with S atoms in the monolayer WSe_2_, we observed similar electrical behavior [109]. The calculated mobilities for the monolayer WSe_2(1−*x*)_S_2*x*_ with *x* = 0.07 and 0.85 were ~0.02 cm^2^·V^−1^·s^−1^,while for the WSe_2(1−*x*)_S_2*x*_ with *x* = 0.28, a much higher mobility of 0.2 cm^2^·V^−1^·s^−1^ was achieved. The mobility difference observed in the WSe_2(1−*x*)_S_2*x*_ alloys might be caused by the different defect densities in the as-grown samples as well as the contact issues. After we coated a layer of 20 nm Al_2_O_3_ on the surface of WSe_2(1−*x*)_S_2*x*_ (*x* = 0.28) as shown in Figure 9d [43], the top-gated device displayed mobility of ∼46.5 cm^2^·V^−1^·s^−1^, two orders of magnitude higher than the back-gated one. Compared to the current prevailing III–V materials, 2D TMDs with low mobility may not be suitable for high-performance applications. However, large band gap and excellent electrostatic integrity make TMDs suitable for applications in low power related areas [172].

### 4.2. Optoelectronic Devices

The wide band gap (1.2–1.9 eV), especially monolayer direct band gap materials, makes TMDs suitable for applications in optoelectronic devices, because of their high absorption coefficient and efficient electron-hole pair generation [21,95,168]. Lopez-Sanchez et al. fabricated a phototransistor based on monolayer MoS_2_ [168]. The schematic view of the photodetector is shown in Figure 9e; the focused laser beam is used to probe the device. The photodetector showed a photoresponse in the range of 400–680 nm and an ultrasensitive behavior with a maximum external photoresponsivity of 880 A·W^−1^ at a wavelength of 561 nm. The direct band gap in monolayer MoS_2_ also resulted in ~9000 fold higher photoresponsivity in monolayer MoS_2_ compared to that in multilayer devices. When the V_ds_ = 8 V and V_g_ = −70 V were applied, the photodevice showed a typical rise time and drop time of τ_rise_ = 4s and τ_decay_ = 9s, respectively. The slow switch between the On state and Off state might be influenced by the surroundings of the MoS_2_ photodevice, like O_2_/H_2_O molecule adsorption [168,173]. The monolayer MoS_2_ based photodetector fabricated by Zhang’s group shows an excellent switching behavior [169]. Figure 9f shows the photoswitching rate test of photoswitching behavior at V_ds_ = 1 V, P_light_ = 80 μW. The observed switching duration for the current rise or decay process was only ~50 ms, much faster than the value reported by Lopez-Sanchez and co-workers [169]. However, the calculated photoresponsivity was very low—could only reach as high as 7.5 mA/W.

The p-n diodes composed of atomically thin TMDs were also realized. In general, the n-type materials referred to MoS_2_, MoSe_2_ or WS_2_, and the p-type material referred to WSe_2_. Cheng et al. fabricated WSe_2_/MoS_2_ heterojunction diodes by vertically stacking the synthetic WSe_2_ and exfoliated MoS_2_; a schematic illustration is depicted in Figure 9g [170]. They observed a clear photovoltaic effect with an open-circuit voltage of ∼0.27 V and a short-circuit current of ∼0.22 μA in the as prepared p-n diodes by measuring its output characteristics (Figure 9h). The external quantum efficiency (EQE) in the heterojunction could reach a maximum of 12% under a 514-nm laser excitation with a power of 0.5 μW. To further increase the EQE of the WSe_2_/MoS_2_ heterojunction diodes, Lee and co-workers prepared a graphene-sandwiched WSe_2_/MoS_2_ heterojunction diodes with different thicknesses [174]. The measured maximum EQE at 532 nm was 34%; this was mainly due to the fact that the graphene with high conductivity could reduce the interlayer recombination of the majority carriers and speed up the collection of the photo-generated carriers. However, the improved value was still inferior to that of materials used in the industry, such as Si, GaAs, CdTe or CuIn*_x_*Ga_(1−*x*)_Se_2_, which showed maximum value of >80% in the visible range [175,176,177,178]. The low EQE in TMDs based photovoltaic devices may be caused by defects and surface states, which are produced during the synthesis and layers’ assembly, respectively. The defects or surface states fasten the carrier recombination or localize the carriers, so that the number of carriers collected by electrodes reduces greatly.

The heterojunction based on 2D TMDs could also be applied to the light emitting diode (LED). Figure 9i showed a schematic illustration of an LED by using MoS_2_ and WSe_2_ as the n-type and the p-type material, respectively [171]. In the LED structure, the MoS_2_ and WSe_2_ were used to inject the electrons and holes, while the sandwiched black phosphorus was used as the trapping site of the injected carriers for light emission.

### 4.3. Hydrogen Evolution Reaction (HER)

Environmental pollution caused by the consumption of non-renewable fossil energy has made the development of new energy a hot topic in the academic society. Electrocatalytic hydrogen production has been considered as one of the most effective ways to produce clean hydrogen energy. So far, Pt and other noble metals were the best electrocatalysts for a hydrogen evolution reaction. However, limited resources and the high price of noble metals restrict their large-scale application. Thus, the search for new catalysts alternative to Pt is imminent. Among the many-layered TMDs, MoS_2_ is the first one to emerge as an active hydrogen evolution reaction catalyst. Bulk MoS_2_ was used as a catalyst for HER in the 1970s, but it showed poor catalytic activity (an onset potential of 90 meV and a Tafel slope of 692 mV/dec) [179]. The rapid development of nanotechnology facilitated the exploration of the performance of nanostructured materials. Contrary to bulk MoS_2_, MoS_2_ nanoparticles were an active HER catalyst, which was demonstrated by Nørskov’s group in 2005 [180]. Since then, two-dimensional nanostructured TMDs have attracted extensive research interest as electrocatalysts for hydrogen evolution [181,182,183,184,185,186]. 

#### 4.3.1. Increasing the Number of Active Sites

**Figure 10:**
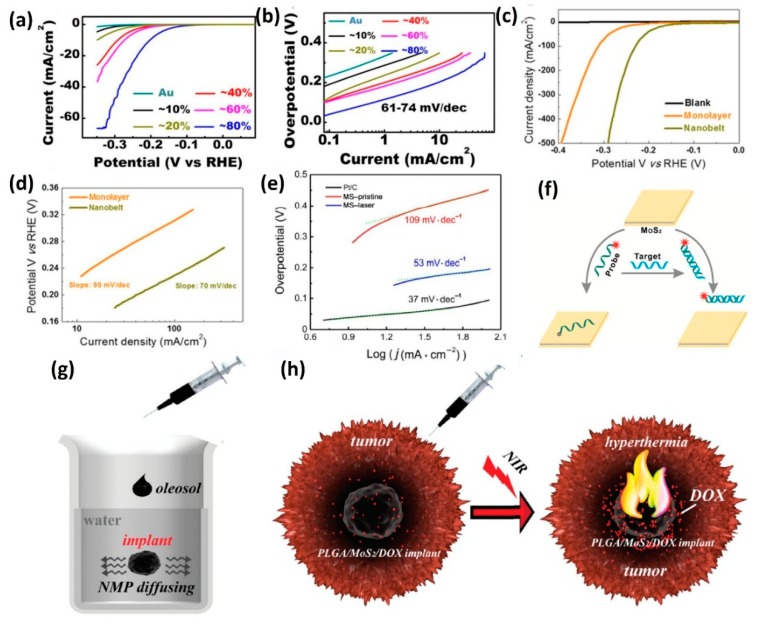
Application of low-dimensional TMDs in HER, hydrogen storage and biosystems. (**a**) Polarization curves of monolayer MoS_2_ on Au foils with different coverage. The 80% coverage sample exhibited current density of ∼50.5 mA/cm^2^ at η = 300 mV, which was much larger than that of the 60%, 40%, 20%, and 10% coverage samples (15.3, 10.1, 5.7and 3.9 mA/cm^2^, respectively) [148]. (**b**) Tafel plots of monolayer MoS_2_ on Au foils with different coverage. The overall Tafel slopes were in the range of 61–74 mV/decade and the lowest Tafel slope was achieved from the sample with ∼80% coverage (61 mV/decade) [148]. (**c**) Polarization curves of monolayer MoS_2_ and MoS_2_ nanobelts. At the current density of 20 mA/cm^2^, the overpotential of monolayer MoS_2_ and MoS_2_ nanobelts were 170 mV and 250 mV, respectively [80]. (**d**) Tafel plots of monolayer MoS_2_ and MoS_2_ nanobelts. The Tafel slope of the nanobelts was 70 mV/decade, which was lower than that of monolayer MoS_2_ (90 mV/decade) [80]. (**e**) Tafel plots of pristine MoS_2_, MoS_2_ QDs, and commercial Pt/C. Compared with the pristine MoS_2_ (109 mV/dec), the MoS_2_ QDs prepared by ultrafast laser ablation showed a much-lower Tafel slope with the value of 53 mV/dec, which was close to the value measured for commercial Pt/C (37 mV/dec) [188]. (**f**) Schematic illustration of the fluorimetric DNA assay. MoS_2_ adsorbed dye-labeled single-stranded DNA probe via the van der Waals force between MoS_2_ and nucleobases, and then quenched the fluorescence of the probe. Compared with single-stranded DNA probe, the interaction between the formed double-stranded DNA and MoS_2_ was weaker, which made the dye-labeled probe away from the surface of MoS_2_. As a result, the fluorescence of the probe was recovered [189]. (**g**) PLGA chains in PMD oleosol undergo an immediate liquid–solid phase transformation on contact with water and the MoS_2_ nanosheets and anticancer drug DOX encapsulates inside the implant matrix [190]. (**h**) Schematic depiction of the PMD oleosol in tumor therapy with NIR laser irradiation. The MoS_2_ showed a high NIR absorbance. With NIR laser irradiation, the generated heat causes significant tumor coagulation necrosis; thus, the tumor can be completely erased without recurrence [190]. Reproduced with permission from [80]. Copyright American Chemical Society, 2015. Reproduced with permission from [148]. Copyright American Chemical Society, 2014. Reproduced with permission from [188]. Copyright Springer, 2017. Reproduced with permission from [189]. Copyright American Chemical Society, 2013. Reproduced with permission from [190]. Copyright Wiley-VCH Verlag GmbH & Co. KGaA, Weinheim, 2015.

Theoretical calculations and experimental results have revealed that the basal plane of TMDs was catalytically inert, whereas the surface edges were chemically active [56,180,187]. Therefore, increasing the number of active sites is an effective way to enhance their catalytic performance. Liu’s group synthesized monolayer MoS_2_ on Au foil substrate by using a CVD method [148]. Monolayer triangular shape MoS_2_ with different coverage (10%, 20%, 40%, 60%, 80%) was obtained upon varying the growth temperature or the precursor-substrate distance. On increasing the coverage of MoS_2_, the average edge length was increased, which was confirmed by representative SEM images. The polarization curves of various samples in Figure 10a indicate that the 80% coverage sample exhibited the lowest onset overpotentials (η) with the value of ∼100 mV. At the same time, the 80% coverage sample showed cathodic current density of ∼50.5 mA/cm^2^ at η = 300 mV, which was much larger than that of 60%, 40%, 20% and 10% samples (15.3, 10.1, 5.7, and 3.9 mA/cm^2^, respectively). As shown in Figure 10b, the overall Tafel slopes measured in the range of 61–74 mV/dec and the 80% coverage MoS_2_ sample showed the lowest Tafel slope (61 mV/decade). The one-dimensional MoS_2_ nanobelts synthesized by our group (details in Section 3.9) had an onset overpotential of −170 mV, lower than that of monolayer MoS_2_ (−250 mV) [80]. Compared with monolayer MoS_2_ (90 mV/dec), MoS_2_ nanobelts showed a smaller Tafel slope (70 mV/dec). The top surface was composed of the edges of the base planes, which provided a high density of edge sites, so that the nanobelts exhibited a more superior catalytic activity than the monolayer MoS_2_ (Figure 10c,d). 

TMDs QDs have a higher special surface area and more edge atom, which should benefit the catalytic activity toward HER. Xu et al. studied the electrocatalytic performance of MoS_2_ and WS_2_ composites (composites of nanosheets and QDs) [191]. The onset overpotential of MoS_2_ and WS_2_ composites were ~120 mV and ~180 mV, respectively. The MoS_2_ and WS_2_ composites prepared in DMF and NMP showed the same onset overpotential, which indicated that the solvent had little or no effect on the chemical property and structure of these QDs. For MoS_2_ and WS_2_ composites, the Tafel slopes ranged from 69 to 75 mV/dec, which were similar to that of MoS_2_ nanobelts. The excellent catalytic activity might result from two factors: (1) The QDs might possess unique defect-rich structure that could bring in more active edge sites; (2) the efficiency of electrons transferred between the active edge sites and electrode was enhanced due to the random or disordered stacking of the exfoliated nanosheets on the surface of glass carbon electrode. Recently, Li’s group successfully fabricated MoS_2_ QDs of about 2 nm diameter by using a facile and general ultrafast laser ablation method [188]. The as-prepared MoS_2_ QDs showed significant lattice deformation and high amounts of surface defects. Owing to their large specific surface area, high content of active sites, good hydrophilicity and high conductivity, the MoS_2_ QDs exhibited high catalytic activity; the overpotential was 108 mV at 10 mA/cm^−2^, and the Tafel slope was 53 mV/dec, as shown in Figure 10e.

#### 4.3.2. Optimizing the Electronic Structure

The Gibbs free energy (ΔG_H_) for hydrogen adsorption on the active sites plays a key role in the catalytic efficiency of a catalyst; the optimal Gibbs free energy for hydrogen adsorption is close to zero [20,56,180,192,193]. The monolayer MoS_2(1−*x*)_Se_2*x*_ alloys with different Se contents mentioned in Section 3.8 had different electronic structures, confirmed by Raman scattering and PL characterizations [107]. The electrocatalytic activity characterizations indicated that in monolayer MoS_2_, the overpotential at the current density of 10 mA/cm^2^ was −335 mV, which was larger than that of monolayer MoS_2(1−*x*)_Se_2*x*_ (−300 mV (*x* = 0.39), −273 mV (*x* = 0.51) and −279 mV (*x* = 0.61)). At the same time, the Tafel slope of monolayer MoS_2(1−*x*)_Se_2*x*_ was 100 mV/dec (*x* = 0.39), 119 mV/dec (*x* = 0.51), 106 mV/dec (*x* = 0.61), smaller than that of pure monolayer MoS_2_ (134 mV/dec). The Gibbs free energy of hydrogen adsorption is associated with the density of states near the Fermi level. Reduced ΔG_H_ can be achieved when the density of states near Fermi level is increased. Compared with pure MoS_2_, the Mo atoms in MoS_2(1−*x*)_Se_2*x*_ alloys have a lower oxidation state. As a consequence, reduced hydrogen adsorption energy was obtained in MoS_2(1−*x*)_Se_2*x*_ alloys. Therefore, enhanced catalytic performance was achieved in the MoS_2(1−*x*)_Se_2*x*_ alloys compared with pure MoS_2_. Similar enhanced catalytic performance was also observed in monolayer WS_2(1−*x*)_Se_2*x*_ alloys [108].

#### 4.3.3. Improving Electrical Conductivity

Increasing electrical transport is another efficient way to increase electrocatalytic performance because high conductivity can effectively facilitate electron transport during the catalytic process. Lukowski and co-workers transformed the MoS_2_ from the semiconductor phase to the metal phase via lithium intercalation [194]. The formation of the 1T-MoS_2_ after lithium intercalation was confirmed by X-ray diffraction, Raman scattering, HRTEM, etc. In an acidic electrolyte, the 2H-MoS_2_ had onset overpotential (at a current density of 10 mA/cm^2^) of −320 mV, and Tafel slope of 110 mV/dec. However in 1T-MoS_2_, as expected, it exhibited enhanced HER activities—onset overpotential was −195 mV vs. RHE, and Tafel slope was 43 mV/dec, respectively. Besides phase transformation, growing low-dimensional TMDs on conductive substrates or fabricating TMDs-based heterostructures can also be used to improve their electrical conductivity [195,196,197,198]. As discussed in Section 3.8, WS_2(1−*x*)_Se_2*x*_ nanotubes were successfully synthesized on conductive CFs [45]. When the WS_2(1−*x*)_Se_2*x*_ nanotubes on CFs served as working electrodes for HER, they exhibited excellent electrocatalytic performance.

### 4.4. Electrochemical Hydrogen Storage

Besides hydrogen production, hydrogen storage is also a challenge in hydrogen technology. As mentioned in Section 3.9, one-dimensional MoS_2_ nanotube could be synthesized by directly heating (NH_4_)_2_MoS_4_ in hydrogen/thiophene atmosphere [158]. Chen et al. examined the electrochemical hydrogen storage property by measuring the cyclic voltamogram, in which the reduction and oxidation process corresponded to the hydrogen adsorption and desorption, respectively. The hydrogen adsorption amount on MoS_2_ nanotubes obtained by the electrochemical method showed the highest, comparing to previous reported work, and the highest capacity was 260 mAh/g (corresponding to the formula of H_1.57_MoS_2_, 0.97 wt.% hydrogen) at 50 mA/g and 20 °C. The high capacity of the MoS_2_ nanotube was attributed to the enhanced electrochemical catalytic activity of its highly nanoporous structure. Meanwhile, they believed that the physicochemical interaction was responsible for the high hydrogen adsorption of MoS_2_ nanotubes. However, further research work is still necessary to understand the precise nature of the interaction between hydrogen and MoS_2_ nanotubes during storage. 

### 4.5. Biosystems

Biomolecular detection plays a key role in the fields of medical diagnostics, drug discovery, food safety, environmental monitoring, and so on. Zhang’s group reported the detection of DNA and small molecules by using MoS_2_ nanosheets as a sensing platform [189]. Their ideas were from findings that the Mo ions in MoS_2_ possessed intrinsic fluorescence-quenching properties and MoS_2_ showed different affinities toward single-stranded DNA versus double-stranded DNA. MoS_2_ adsorbed dye-labeled single-stranded DNA probe via the van der Waals force between MoS_2_ and nucleobases, and then quenched the fluorescence of the probe. When a single-stranded DNA probe was hybridized with its complementary target DNA, the double-stranded DNA was formed. Compared to single-stranded DNA probe, interaction between the formed double-stranded DNA and MoS_2_ was weaker, which led the dye-labeled probe away from the surface of MoS_2_. As a result, the fluorescence of the probe was recovered and was used to read the quantity of the target DNA. Figure 10f schematically describes the whole process.

The TMDs can accommodate a large mass of drugs, which offers great potential in pharmaceutical applications [96]. For example, they could act as drug carriers and deliver the drugs to the specified cells. As drug carriers, drug-loading capability is a very important parameter [96]. For 2D TMDs, the highest-reported drug loading ratio (weight ratios between the drug and 2D TMDs) were determined as ≈240%, higher than that of graphene oxide (≈150%). Moreover, 2D TMDs also show a high near infra-red (NIR) absorbance, which make them suitable for photo thermal ablation of diseases such as tumors [98]. For localized tumor-synergistic therapy, Wang et al. prepared the phase-changing organic-inorganic PLGA/MoS_2_/DOX composite oleosol by homogenizing PLGA, MoS_2_ and DOX together into the NMP solvent, where the PLGA referred to Poly (lactic- co -glycolic acid), and the DOX referred to doxorubicin [190]. When the PLGA/MoS_2_/DOX oleosol came in contact with body fluid, unlike the traditional drug/carrier manner of “dispersed-in-suspension”, the oleosol would immediately solidify (Figure 10g), and the MoS_2_ nanosheets and anticancer drug DOX was encapsulated inside the implant matrix. Therefore, a solid implant with high potential in synergistic tumor photo thermal and chemotherapy was formed. With NIR laser irradiation, the generated heat causes significant tumor coagulation necrosis; thus, the tumor can be completely erased without recurrence. The schematic process is shown in Figure 10h. 

TMDs QDs could also be used in cancer radiation therapies [139]. Experimental investigations indicate that the cysteine-protected WS_2_ QDs exhibit strong catalytic activities toward the reduction of H_2_O_2_ and O_2_, and showed excitation-wavelength dependent fluorescence. These properties made the WS_2_ QDs a potential candidate to improve cell viabilities and eliminate reactive oxygen species in injured cells. In vivo studies showed that the WS_2_ QDs could also prevent DNA damages in irradiated cells under exposure to high-energy gamma rays. At the same time, the cysteine-protected WS_2_ QDs could recover superoxide dismutase and remove excessive Methylenedioxyamphetamine in the liver and lung by participating in the catalytic processes and omitting reactive oxygen species. Furthermore, nearly 80% of WS_2_ QDs was rapidly excreted through renal routes 1 day post injection and no notable toxicological responses was found up to 30 days at a relatively high injection dose.

## 5. Summary and Outlook

This review has summarized recent developments in the preparation of low-dimensional TMDs. Several methods such as mechanical cleavage method, liquid exfoliation method, Li-intercalation exfoliation method, laser and plasma-induced thinning method, CVD method and so on have been adopted to prepare low-dimensional TMDs. In view of their fascinating properties and diverse preparation methods, low-dimensional TMDs have been utilized in many ways, such as devices, energy, biosystems, etc. Although significant achievements have been made in low-dimensional TMDs related areas, there were several problems. Taking MoS_2_ as an example, the room-temperature top and back gated configuration field-effect mobility were ~200 cm^2^·V^−1^·s^−1^ and ~1.2 cm^2^·V^−1^·s^−1^, respectively, which was much lower than that of silicon (~1400 cm^2^·V^−1^·s^−1^). To replace silicon in semiconductor chips, further improvement of the electrical performance of TMDs-based devices is necessary. TMDs-based catalysts have exhibited excellent electrocatalytic performance, although their HER activities were still inferior to noble metals like Pt. Thus, further enhancement of the electrocatalytic performance of TMDs is essential and imperative. Mass production of high-quality TMDs remains challenging because the size of a single crystal of TMDs obtained from the methods mentioned above is about dozens of microns, which is insufficient for industrial applications. As discussed in Section 4.3, the EQE of TMDs based diodes was still very low, compared to materials used in the industry. Ways to increase EQE should concentrated on improving the quality of materials and optimizing the interface, because the physical transfer and sequential chemical synthesis of the initial layers can damage the intrinsic structure and influence the physical properties of the layers. 

Theoretical calculations and experimental results indicate that the plane edges of semiconductor TMDs are metallic, but the electronic transport properties and their application in devices is still unknown. To gain a better understanding of the electronic transport performance of TMDs, it is worth specializing in the synthesis of TMDs with totally exposed metallic edges and fabrication of metallic edges-based FET. Meanwhile, it is meaningful to fabricate hetero-dimensional nanostructures with TMDs nanowires metallic edges that have been contacted with monolayer TMDs, forming FET with metallic edge electrodes. Studies on contact resistance between the source/drain electrodes and the channel and the performance of the FET are also a possibility.

Diluted magnetic semiconductors (DMSs) have attracted great interest because they combine two large branches in one material—the charge degree of freedom of electrons in semiconductors and the spin degree of freedom in magnetic materials. The coexistence of ferromagnetism and semiconducting properties can be realized by introducing magnetic elements into nonmagnetic semiconductors. At present, studies on DMSs are centered mostly on III-V and II-VI based DMSs, such as (Cd, Mn)Te, (Zn, Mn)S, (In, Mn)As and (Ga, Mn)As. However, TMDs-based DMSs have never been involved. The successful synthesis of few-layer TMDs alloying with magnetic elements will enrich the DMSs family and trigger fascinating properties due to their special structure. 

## Figures and Tables

**Table 1:** Frequencies of $E_{2g}^{1}$ and *A*_1*g*_ Raman modes and their difference with different layer thickness.

Thickness (L)	$E_{2g}^{1}$ (cm^−1^)	*A*_1*g*_ (cm^−1^)	Frequency Difference(cm^−1^)
1	~384.4	~403.0	~18.6
2	~383.3	~404.8	~21.5
3	~382.7	~405.8	~23.1
4	~382.3	~406.7	~24.4
5	~382.3	~406.9	~24.6
6	~382.2	~407.0	~24.8
Bulk	~382.0	~407.4	~25.4
